# The effects of the recurrent social isolation stress on fear extinction and dopamine D_2_ receptors in the amygdala and the hippocampus

**DOI:** 10.1007/s43440-022-00430-8

**Published:** 2022-11-17

**Authors:** Aleksandra Wisłowska-Stanek, Małgorzata Lehner, Filip Tomczuk, Aleksandra Gawryluk, Karolina Kołosowska, Anna Sułek, Paweł Krząśnik, Alicja Sobolewska, Adriana Wawer, Adam Płaźnik, Anna Skórzewska

**Affiliations:** 1 Department of Experimental and Clinical Pharmacology, Centre for Preclinical Research and Technology (CEPT), Medical University of Warsaw, 1B Banacha Street, 02-097 Warsaw, Poland; 2 Department of Neurochemistry, Institute of Psychiatry and Neurology, 9 Sobieskiego Street, 02-957 Warsaw, Poland; 3 Department of Genetics, Institute of Psychiatry and Neurology, 9 Sobieskiego Street, 02-957 Warsaw, Poland; 4 Laboratory of Neuroplasticity, Nencki Institute of Experimental Biology of Polish Academy of Sciences, 3 Pasteur Street, 02-093 Warsaw, Poland

**Keywords:** Social isolation, Fear extinction, Dopamine D_2_ receptors, Amygdala, Hippocampus

## Abstract

**Background:**

The present study assessed the influence of recurrent social isolation stress on the aversive memory extinction and dopamine D_2_ receptors (D_2_R) expression in the amygdala and the hippocampus subnuclei. We also analyzed the expression of epigenetic factors potentially associated with fear extinction: miRNA-128 and miRNA-142 in the amygdala.

**Methods:**

Male adult fear-conditioned rats had three episodes of 48 h social isolation stress before each fear extinction session in weeks intervals. Ninety minutes after the last extinction session, the D_2_R expression in the nuclei of the amygdala and the hippocampus (immunocytochemical technique), and mRNA levels for D_2_R in the amygdala were assessed (PCR). Moreover, we evaluated the levels of miRNA-128 and miRNA-142 in the amygdala.

**Results:**

It was found that recurrent social isolation stress decreased the fear extinction rate. The extinguished isolated rats were characterized by higher expression of D_2_R in the CA1 area of the hippocampus compared to the extinguished and the control rats. In turn, the isolated group presented higher D_2_R immunoreactivity in the CA1 area compared to the extinguished, the control, and the extinguished isolated animals. Moreover, the extinguished animals had higher expression of D_2_R in the central amygdala than the control and the extinguished isolated rats. These changes were accompanied by the increase in miRNA-128 level in the amygdala in the extinguished isolated rats compared to the control, the extinguished, and the isolated rats. Moreover, the extinguished rats had lower expression of miRNA-128 compared to the control and the isolated animals.

**Conclusions:**

Our results suggest that social isolation stress impairs aversive memory extinction and coexists with changes in the D_2_R expression in the amygdala and hippocampus and increased expression of miRNA-128 in the amygdala.

**Supplementary Information:**

The online version contains supplementary material available at 10.1007/s43440-022-00430-8.

## Introduction

Conditioned fear extinction is learned inhibition of retrieval of the previously acquired fear response. It represents the formation of a new memory that inhibits conditioned–unconditioned stimulus contingency when the conditioned stimulus is presented without an unconditioned stimulus [1]. Extinction is essential for adaptive processes and the regulation of negative emotions [2]. The impairment of fear extinction is associated with the pathogenesis of anxiety disorders, such as posttraumatic stress disorder (PTSD), phobias, and obsessive–compulsive disorder. It is often used as a component of exposure therapy in patients with anxiety disorders [3–6]. Social isolation has often been associated with an impaired stress response, increased attention to negative stimuli, and impaired fear extinction leading to a heightened predisposition to affective and anxiety disorders [7–9].

Amygdala is a crucial structure that plays an essential role in fear extinction [10]. Accumulating evidence indicates that the basolateral nucleus, especially the lateral nucleus (LA) of the amygdala, plays an essential role in extinction [10, 11]. The basolateral amygdala receives information on aversive stimuli and sends them to the central nucleus of the amygdala (CeA) [12]. The CeA evokes behavioral fear responses via its projection to the hypothalamus and brainstem [12]. In turn, the hippocampus, especially its dorsal part, is essential for processing contextual information and affects the amygdala function [13–15].

Dopamine D2 receptors (D_2_R) in the amygdala are vital in regulating aversive fear memories and anxiety processing [16–18]. Stressful events may negatively regulate the dopaminergic system and induce anxiety or depression-like behaviors [19, 20]. Our previous results revealed that restraint stress increased the dopaminergic neurotransmission in the amygdala and induced depression-like behavior (decreased sucrose preference) [21]. Similarly, chronic social isolation stress in Wistar rats increased dopamine metabolite 3-methoxytyramine levels in the amygdala [22]. Preclinical and clinical studies suggest that the dopaminergic system modulation may enhance fear extinction: e.g., it was found that methylphenidate, MDMA (3,4-methylenedioxymethamphetamine), and L-DOPA increased the extinction rate [20, 23, 24].

Considering that memory processes depend on genetic and environmental factors, we would like to assess the effects of social isolation stress on the fear extinction and expression of D2R in the hippocampus and amygdala as well as epigenetic factors associated with fear extinction like miRNA-128 and miRNA-142 [25–29]. Previously, it was found that a change in miRNA-128 level may be associated with depression-like behavior and passive coping strategies in stress conditions [30]. Here, we assessed the D_2_R expression in distinct subnuclei of the amygdala and in the hippocampus, as little is known about their involvement in fear extinction upon stress conditions.

## Materials and methods

### Animals

Experiments were performed on 40 male, adult 9-weeks-old Wistar rats weighing 220–250 g at the beginning of the experiments. The animals were housed 5 in an opaque plastic cage in standard laboratory conditions (temperature 20 ± 2 °C; 12 h light/dark cycle, light on at 7 am; 45–55% humidity) with ad libitum access to water and rodent chow. The cages were enriched with wood for gnawing. The study was conducted under the European Communities Council Directive 2010/63/UE. The Local Committee for Animal Care and Use at the Medical University of Warsaw, Poland, approved this study (Protocol No. 34/2015).

### Experiment protocol (Fig. 1)

**Fig. 1:**
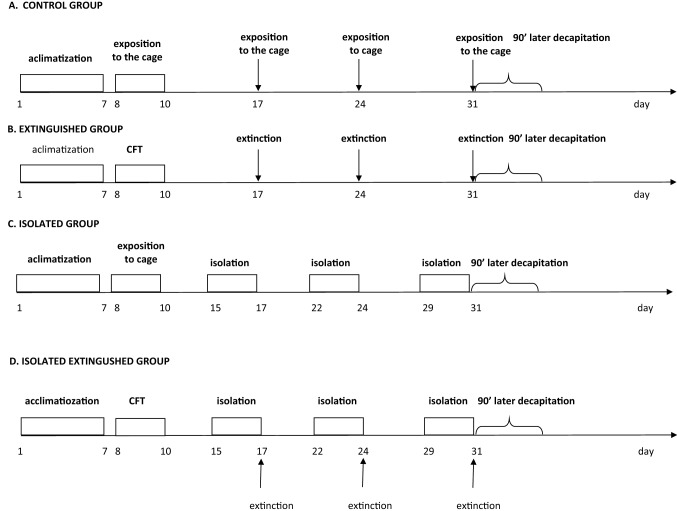
The experimental scheme. The influence of intermittent social isolation on fear extinction. *CFT* conditioned fear test; isolation—48 h of social isolation; extinction—fear extinction

Four experimental groups: (1) control, (2) extinguished, (3) isolated, and (4) isolated extinguished group, were used in the study. After 7 days of acclimatization to the vivarium, on the experiment’s 8, 9 and 10th day, the extinguished group and the isolated extinguished group underwent fear conditioning, while the control and the isolated groups received no unconditioned stimulus only were exposed to the experimental cage.

Next, three times (on the 17th, 24th, and 31st day of the experiment), previously conditioned animals were exposed to three extinction sessions lasting 10 min. At the same time, the control animals were exposed to the experimental cage. The animals were socially isolated for 48 h before each extinction session. The animals were decapitated on the 31st day, 90 min after exposition to the experimental cage. The scheme of the experimental protocol is shown in Fig. 1.

### Conditioned fear test (CFT)

The fear conditioning was performed in experimental cages under constant white noise conditions for 3 consecutive days. On the first day, the animals were individually placed in the box for 2 min to adapt to the experimental conditions. On the second day, after 5 min of habituation, the animals underwent a fear conditioning procedure that consisted of three footshocks (stimulus 0.7 mA, 1 s, repeated every 59 s). Conditioned fear was tested on the third day by re-exposing the rats to the testing box and recording their freezing response over 10 min (freezing was measured through fear conditioning software, TSE, Bad Homburg, Germany) [31].

### Extinction sections

Three extinction sessions were performed weekly, the first—a week after the test day of conditioned fear. During extinction sessions, the rats’ freezing time was examined for 10 min in the testing box (exposure to aversive context) [31].

### Social isolation stress

48 h before each extinction session, the rats from the isolated groups were socially isolated. These rats were separated and housed individually in non-transparent cages (cage size, 36 × 27 × 19 cm) in the vivarium with food and water provided ad libitum [32].

### Biochemical analysis

The rats were decapitated 90 min after the third extinction session. Their brains were removed and divided into two hemispheres. One hemisphere was frozen in dry-ice cooled cyclopentane, stored at − 70 °C, and then used for immunocytochemistry. The amygdala was dissected from the other hemisphere with microanalytical instruments in anatomical borders.

#### Immunocytochemistry

The immunocytochemical technique was performed as previously published [21, 34]. Coronal 20 μm cryostat sections, identified according to the rat brain atlas [33], were cut, mounted on silane-coated slides, and fixed in methanol. Two sections of the brain region were taken for D_2_R immunostaining. After blocking endogenous peroxidase activity and non-specific binding, tissue samples were incubated with primary rabbit polyclonal antibodies against the D2R (1:2000, Abcam) at 4–8 °C for 72 h. Then, the staining levels were detected with peroxidase-conjugated anti-rabbit IgG (1:2000, ImmunoJackson Research). D2R immunoreactivity was examined in the section at AP (− 3.30) for hippocampus areas: DG, CA1, and CA3 and the amygdala: the basal (BA), central (CeA), and lateral nucleus of the amygdala (LA). Immunopositive complexes were manually counted using an image analysis system (Olympus BX-51 microscope with Camera DP 70, Olympus cellSens software). The examined areas were sampled using a 0.15 mm^2^ frame. Two images from each session were taken, and the results from each animal were averaged. The values were expressed as the number of positive cells per mm^2^ [21, 34].

#### Real-time PCR

The amygdala was dissected: AP (−) 2.60 to (−) 3.30 mm based on the rat brain atlas of Paxinos and Watson [33]. The total RNA was extracted and purified as previously described [34]. All samples had Abs 260/280 > 1.9 and 260/230 > 1.4. The quality of the total RNA was further verified using the Agilent 2100 Bioanalyzer (Agilent RNA 6000 Pico Kit; Agilent Technologies).

The analysis of mRNA and miRNA was performed according to the previously described method [34]. Real-time PCR analysis was done using PikoReal™ Real-Time PCR System (Thermo Fisher Scientific) with PowerSYBR^®^ Green PCR Master Mix (Applied Biosystems), specific primers for D2R gene (5′ → 3′, F:CAACAATACAGACCAGAATGAG; R:CAGCAGAGTGACGATGAA), and cDNA (concentration 5 ng/μl) for each sample in the total volume of 10 μl. The housekeeping reference gene is glyceraldehyde-3-phosphate dehydrogenase (GAPDH, 5′ → 3′, F:ATGACAATGAATATGGCTACA; R:CTCTTGCTCTCAGTATCCTT). From samples of cDNA, real-time PCR was conducted for mRNA for D2R. The amplification reaction included 40 cycles with a 95 °C denaturation step for 5 s and a 61 °C annealing step for 45 s. A dissociation stage was performed to assess the specificity of primers. Each sample was run in triplicate. Real-time PCR assays of total RNA were performed to measure the expression levels of miR-128 and miR-142-3p in the amygdala. Relative levels were normalized to U6 snRNA and 4.5S RNA(H). Analysis of miRNAs was done using LightCycler 480 Real-Time PCR System (Roche) with TaqMan^®^ Universal Master Mix II, no UNG (Applied Biosystems), specific TaqMan^®^ Probes (TaqMan™ miRNA Assays, Termofisher Scientific), and products of reverse transcription. Each sample was run in triplicate. Analysis of all real-time PCR data was performed using the comparative ΔΔCT method [34].

### Statistical analysis

All data are shown as the means + standard error of the mean (SEM). The Shapiro–Wilk test assessed data normality, and all data presented had a normal distribution. The behavioral data were analyzed by repeated measures ANOVA. Biochemical data were analyzed by two-way ANOVA. All analyses were followed by Newman–Keuls post hoc test. All statistical analyses were performed using Statistica v.12 software.

## Results

### Behavioral data (Fig. 2)

**Fig. 2:**
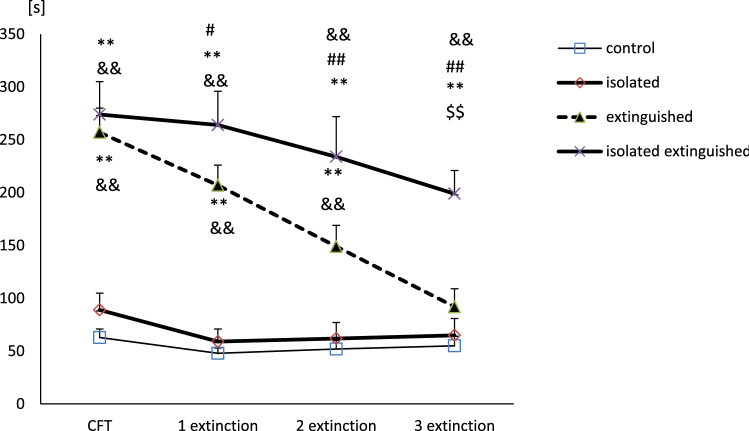
The effect of social isolation stress on fear extinction in rats (freezing time during 10 min exposition to aversive context). Extinction sessions were performed in 1 week intervals. The first one was performed 1 week after the test session of fear conditioning. Control—the control rats (*n* = 10), extinguished—the extinguished rats (*n* = 10), isolated—the isolated rats (*n* = 10); isolated extinguished—the isolated extinguished rats (*n* = 10), and CFT—conditioned fear test. **, *- differs from the control rats, *p* < 0.01; *p* < 0.05; ^&&^—differs from the isolated rats, *p* < 0.01; ^##,#^—differs from CFT (conditioned fear test) session the extinguished group, *p* < 0.01, *p* < 0.05, ^$$^—differs from the extinguished rats, *p* < 0.01. Repeated-measures ANOVA, followed by Newman–Keuls post hoc. The data are shown as the means + SEM

Two-way repeated-measure ANOVA showed significant differences in the freezing time among groups for: the freezing effect (*F*_1,36_ = 72.07; *p* < 0.01), isolation effect (*F*_1,36_ = 5.14; *p* < 0.05); time effect (*F*_3,108_ = 20.31; *p* < 0.01), freezing x time effect (*F*_3,108_ = 12.90; *p* < 0.01); time × freezing × isolation effect (*F*_3,108_ = 2.70; *p* < 0.05); no freezing x isolation effect (*F*_1,36_ = 2.41; *p* = 0.12); isolation × time effect (*F*_3,108_ = 1.72; *p* = 0.16). Newman–Keuls post hoc test showed significantly longer freezing time in extinguished and isolated extinguished rats compared to control rats (*p* < 0.01) and isolated animals during test sessions of CFT, first and second extinction (*p* < 0.01). During the third extinction session, only isolated extinguished rats had a longer freezing time compared to control rats (*p* < 0.01). Moreover, it was found that extinguished rats during the conditioned test session had significantly longer freezing time compared to first, second, and third extinction sessions (*p* < 0.05; *p* < 0.01 and *p* < 0.01, respectively). Additionally, during the third extinction session, isolated extinguished rats had significantly longer freezing time compared to extinguished and isolated rats (*p* < 0.01).

### Biochemical data

#### Immunocytochemical expression of D2R (Fig. 3)

**Fig. 3:**
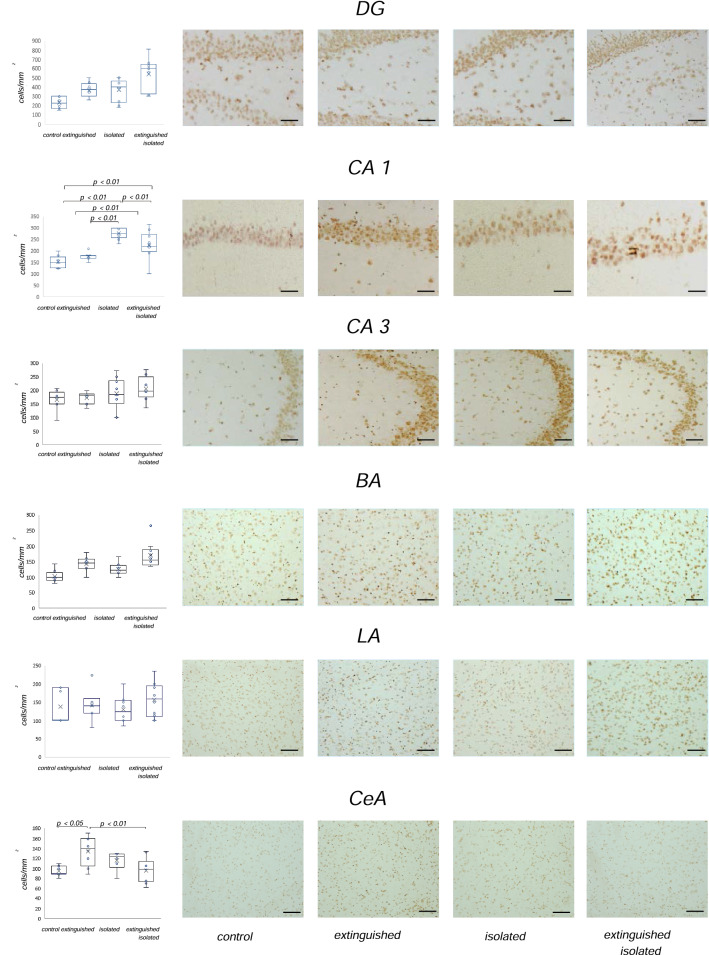
Expression of dopamine receptors (D_2_R) in the hippocampus and the amygdala in rats after the third extinction session was assessed by immunocytochemical staining. Left panel: diagram presenting density of D_2_R immunoreactive neurons (number of cells/mm2). Right panel: photomicrographs showing representative expression of D2R in the dentate gyrus (DG), CA1 and CA3 area of the hippocampus, basal nucleus (BA), lateral nucleus (LA), and central nucleus (CeA) of the amygdala with an objective lens at 20 × magnification (total magnification of 200×). Scale bar indicates 120 µm. Control (*n* = 10), extinguished (*n* = 10), isolated (*n* = 10); isolated extinguished (*n* = 10). Statistics: two-way ANOVA followed by Newman–Keuls post hoc. Data are presented as the mean number of cells per 1 mm2 ± SEM

Two-way ANOVA revealed the significant differences in D_2_R expression in the following structures: **DG: **freezing effect (*F*_1,32_ = 10.78; *p* < 0.01); isolation effect (*F*_1,32_ = 8.63; *p* < 0.01) and no freezing × isolation interaction effect (*F*_1,32_ = 0.19; *p* = 0.66); **CA1:** isolation effect (*F*_1,32_ = 47.00; *p* < 0.01); freezing × isolation interaction effect (*F*_1,32_ = 9.13; *p* < 0.01) and no freezing effect (*F*_1,32_ = 1.15; *p* = 0.29); **BA: **freezing effect (*F*_1,30_ = 18.32; *p* < 0.01); isolation effect (*F*_1,30_ = 6.68; *p* < 0.05); no freezing isolation effect (*F*_1,30_ = 0.04; *p* = 0.83); **CeA**: freezing × isolation effect (*F*_1,27_ = 12.71; *p* < 0.01), no freezing effect (*F*_1,27_ = 1.34; *p* = 0.25), no isolation effect (*F*_1,27_ = 1.04; *p* = 0.31).

There were no significant differences of the D_2_R expression in: CA3: no freezing effect (*F*_1,29_ = 1.03; *p* = 0.31), no isolation effect (*F*_1,29_ = 3.42; *p* = 0.07), no freezing x isolation effect (*F*_1,29_ = 0.0005; *p* = 0.98) and LA: no freezing effect (*F*_1,28_ = 0.99; *p* = 0.32), no isolation effect (*F*_1,28_ = 0.05; *p* = 0.81), and no interaction freezing × isolation effect (*F*_1,28_ = 0.49; *p* = 0.48).

Newman–Keuls post hoc showed: significantly higher expression in the CA1 of isolated extinguished rats compared to the control group (*p* < 0.01) and extinguished rats (*p* < 0.05). Moreover, isolated animals had higher D_2_R expression in CA1 compared to extinguished ones, extinguished isolated, and control rats (*p* < 0.01). In the CeA, extinguished rats had higher D_2_R expression compared to control (*p* < 0.05) and extinguished isolated animals (*p* < 0.01).

#### mRNA level for D_2_R in the amygdala (Fig. 4)

**Fig. 4:**
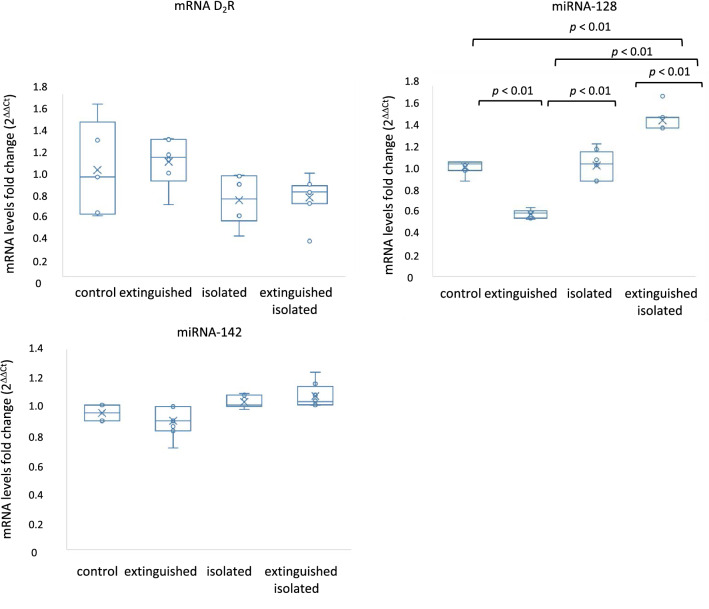
A The D_2_R–dopamine D_2_ receptor mRNA levels. Control (*n* = 7); extinguished (*n* = 8); isolated (*n* = 7); isolated extinguished (*n* = 8); and miRNA levels: B the miRNA-128 levels, control (*n* = 7); extinguished (*n* = 8); isolated (*n* = 7); isolated extinguished (*n* = 8); C the miRNA-142 levels, control (*n* = 7); extinguished (*n* = 8); isolated (*n* = 7); isolated extinguished (*n* = 8) in the amygdala. The mRNA and miRNA levels were measured via PCR and expressed as ΔΔCT. Statistics: two-way ANOVA followed by Newman–Keuls post hoc. Data are presented as the means ± SEM

Two-way ANOVA showed significant differences in the mRNA level for D2R: isolation effect (*F*_1,21_ = 7.58; *p* < 0.05), no freezing effect (*F*_1,21_ = 0.2; *p* = 0.65), and no freezing × isolation effect (*F*_1,21_ = 0.05; *p* = 0.82).

#### miRNA levels in the amygdala (Fig. 4)

Two-way ANOVA revealed altered levels in the amygdala for miRNA-128: isolation effect (*F*_1,25_ = 149.22; *p* < 0.01); freezing x isolation interaction effect (*F*_1,25_ = 141.34; *p* < 0.01); no freezing effect (*F*_1,21_ = 0.079; *p* < 0.05) and miRNA-142: freezing effect (*F*_1,23_ = 4.76; *p* < 0.05); isolation effect (*F*_1,23_ = 11.44; *p* < 0.01); no freezing x isolation interaction effect (*F*_1,23_ = 0.68; *p* = 0.41). Post hoc showed decreased level of miRNA-128 in extinguished compared to isolated and control rats (*p* < 0.01). Moreover, extinguished isolated rats had higher expression of miRNA-128 than other groups of rats (*p* < 0.01).

## Discussion

Our results showed that recurrent social isolation stress 48 h before each extinction session was associated with prolonged freezing time during the third extinction session in socially isolated animals compared to the extinguished group. Simultaneously, during the third extinction session, there was no difference in freezing time between the extinguished and the control rats. On the molecular level, we found that social isolation stress was associated with an increased D_2_R expression in the CA1 area of the hippocampus. Simultaneously, the extinguished animals presented higher D_2_R expression in the central amygdala compared to the control and the extinguished isolated animals. Moreover, we found changes in miRNA-128 after the third extinction session—extinguished rats had lower levels than the isolated, isolated extinguished, and control animals.

Social isolation is a potent stress factor influencing behavioral reactivity to environmental stimuli [9, 10, 35]. The prolonged social isolation stress may cause cognitive disturbances, depressive, or anxiety behavior [10, 36–41]. Moreover, social isolation was shown to cause delayed and incomplete fear extinction [10, 37, 38]. The current study confirmed that even intermittent social isolation before extinction sessions impairs fear extinction.

Our results also confirmed the important role of the dopaminergic neurotransmission in the amygdala and the hippocampus in fear extinction. We found that exposition to an aversive context during the third extinction session was associated with changes in D_2_R expression in the hippocampus and the amygdala in a subnuclei-dependent manner. Our results may partly explain conflicting evidence concerning the D_2_R agonists and antagonists' effectiveness in preclinical models of fear extinction and exposure therapy of anxiety disorders. The discrepancies could be linked to the different roles of distinct amygdalar and hippocampal nuclei in fear extinction [8, 42]. Based on our current results, we could assume that impaired fear extinction is related to increased expression of D_2_R in the CA1 area of the hippocampus in the isolated extinguished rats compared to the extinguished group. Similar changes were observed in the amygdala and concerned the expression of miRNA-128. This coincidence probably reflects the existence of hippocampal–amygdalar interdependencies that could affect the extinction of fear expression in isolated extinguished animals. The changes in the CA1 area of the hippocampus are similar to previous research that indicated the important role of D_2_R in the extinction of appetitive stimuli [43].

In our study, we also analyzed the expression of two miRNAs: miRNA-128 and miRNA-142, which affect fear extinction. Our study showed that the upregulation of the miRNA-128 in the amygdala may be related to memory disruption in socially isolated animals. MicroRNAs are small non-coding RNAs of 19–25 nucleotides in length and are known to regulate several protein-coding genes [44]. In our study, isolated animals characterized by extinction deficits had increased—upregulated levels of miRNA-128 in the amygdala. Similarly, in earlier studies, rodents in the neurodegenerative Huntington’s disease model had upregulated expression of miRNA-128 in the brain [44]. An interesting observation was revealed by Zhou et al., who showed that miRNA-128 reduce apoptosis of dopaminergic neurons, so we could speculate that the increase in miRNA-128 level could have a protective effect on dopaminergic neurons [27].

## Limitations

It is worth emphasizing that the presented results are only observational. Studies on activation and blocking of their function would be more appropriate to confirm the direct role of D_2_R and specific miRNA in extinction processes.

## Conclusions

Our results confirmed the deteriorated effect of intermittent social isolation stress on fear extinction. We showed that social isolation was associated with changes in dopaminergic neurotransmission in extinguished rats, and it increased D_2_R expression in the hippocampus and decreased in the central amygdala. Moreover, we found upregulated miRNA-128 levels in the amygdala of the socially isolated extinguished rats.

## Supplementary Information

Below is the link to the electronic supplementary material.

Supplementary file1 (TIF 29680 KB)

Supplementary file2 (TIF 28033 KB)

Supplementary file3 (TIF 27980 KB)

Supplementary file4 (TIF 26376 KB)

Supplementary file5 (TIF 17919 KB)

Supplementary file6 (TIF 40613 KB)

Supplementary file7 (TIF 25097 KB)

Supplementary file8 (TIF 24081 KB)

Supplementary file9 (TIF 29241 KB)

Supplementary file10 (TIF 25981 KB)

Supplementary file11 (TIF 27647 KB)

Supplementary file12 (TIF 26288 KB)

Supplementary file13 (TIF 26101 KB)

Supplementary file14 (TIF 28155 KB)

Supplementary file15 (TIF 27368 KB)

Supplementary file16 (TIF 29186 KB)

Supplementary file17 (TIF 17845 KB)

Supplementary file18 (TIF 25502 KB)

Supplementary file19 (TIF 26149 KB)

Supplementary file20 (TIF 20142 KB)

Supplementary file21 (TIF 25877 KB)

Supplementary file22 (TIF 13394 KB)

Supplementary file23 (TIF 26950 KB)

Supplementary file24 (TIF 31775 KB)

Supplementary file25 (TIF 26795 KB)

Supplementary file26 (TIF 20646 KB)

Supplementary file27 (TIF 29526 KB)

Supplementary file28 (TIF 24610 KB)

Supplementary file29 (TIF 28392 KB)

Supplementary file30 (TIF 25155 KB)

Supplementary file31 (TIF 25582 KB)

Supplementary file32 (TIF 28313 KB)

Supplementary file33 (TIF 26329 KB)

Supplementary file34 (TIF 23250 KB)

## Data Availability

The data sets generated during and/or analyzed during the current study are available from the corresponding author upon reasonable request.

## Abbreviations

BA: Basal nucleus of the amygdala
CA1, CA3: Cornu ammonis areas of the hippocampus
CeA: Central nucleus of the amygdala
CFT: Conditioned fear test
D_2_R: Dopamine D_2_ receptor
DG: Dentate gyrus of the hippocampus
GAPDH: Glyceraldehyde-3-phosphate dehydrogenase
LA: Lateral nucleus of the amygdala
L-DOPA: L-3,4-dihydroxy-phenylalanine
MDMA: 3,4-Methylenedioxymethamphetamine
PTSD: Posttraumatic stress disorder
SEM: Standard error of the mean

## References

[1] Maren S (2011). Seeking a spotless mind: extinction, deconsolidation, and erasure of fear memory. Neuron.

[2] Pace-Schott EF, Germain A, Milad MR (2015). Effects of sleep on memory for conditioned fear and fear extinction. Psychol Bull.

[3] Furini C, Myskiw J, Izquierdo I (2014). The learning of fear extinction. Neurosci Biobehav Rev.

[4] Marshall PR, Bredy TW (2019). Neuroepigenetic mechanisms underlying fear extinction: emerging concepts. Psychopharmacology.

[5] Maren S, Holmes A (2016). Stress and fear extinction. Neuropsychopharmacology.

[6] Singewald N, Holmes A (2019). Rodent models of impaired fear extinction. Psychopharmacology.

[7] Cacioppo JT, Hawkley LC, Norman GJ, Berntson GG (2011). Social isolation. Ann N Y Acad Sci.

[8] Pibiri F, Nelson M, Guidotti A, Costa E, Pinna G (2008). Decreased corticolimbic allopregnanolone expression during social isolation enhances contextual fear: a model relevant for posttraumatic stress disorder. Proc Natl Acad Sci USA.

[9] Skelly MJ, Chappell AE, Carter E, Weiner JL (2015). Adolescent social isolation increases anxiety-like behavior and ethanol intake and impairs fear extinction in adulthood: possible role of disrupted noradrenergic signaling. Neuropharmacology.

[10] Herry C, Ciocchi S, Senn V, Demmou L, Luthi A (2008). Switching on and off fear by distinct neuronal circuits. Nature.

[11] Barad M, Gean PW, Lutz B (2006). The role of the amygdala in the extinction of conditioned fear. Biol Psychiatry.

[12] Sah P, Westbrook RF (2008). Behavioural neuroscience: the circuit of fear. Nature.

[13] Myers KM, Davis M (2007). Mechanisms of fear extinction. Mol Psychiatry.

[14] Bazaz A, Ghanbari A, Vafaei AA, Khaleghian A, Rashidy-Pour A (2022). Oxytocin in dorsal hippocampus facilitates auditory fear memory extinction in rats. Neuropharmacology.

[15] Radwanska K, Schenatto-Pereira G, Ziółkowska M, Łukasiewicz K, Giese KP (2015). Mapping fear memory consolidation and extinction-specific expression of JunB. Neurobiol Learn Mem.

[16] Bouchet CA, Miner MA, Loetz EC, Rosberg AJ, Hake HS, Farmer CE (2018). Activation of nigrostriatal dopamine neurons during fear extinction prevents the renewal of fear. Neuropsychopharmacology.

[17] Dubovyk V, Manahan-Vaughan D (2019). Gradient of expression of dopamine D2 receptors along the dorso-ventral axis of the hippocampus. Front Synaptic Neurosci.

[18] Dadkhah M, Abdullahi PR, Rashidy-Pour A, Sameni HR, Vafaei AA (2018). Infralimbic dopamine D2 receptors mediate glucocorticoid-induced facilitation of auditory fear memory extinction in rats. Brain Res.

[19] De Bundel D, Zussy C, Espallergues J, Gerfen CR, Girault JA, Valjent E (2016). Dopamine D2 receptors gate generalization of conditioned threat responses through mTORC1 signaling in the extended amygdala. Mol Psychiatry.

[20] Jager A, Kanters D, Geers F, Buitelaar JK, Kozicz T, Glennon JC (2019). Methylphenidate dose-dependently affects aggression and improves fear extinction and anxiety in BALB/cJ mice. Front Psychiatry.

[21] Wisłowska-Stanek A, Płaźnik A, Kołosowska K, Skórzewska A, Turzyńska D, Liguz-Lęcznar M (2019). Differences in the dopaminergic reward system in rats that passively and actively behave in the Porsolt test. Behav Brain Res.

[22] Krupina NA, Khlebnikova NN, Narkevich VB, Naplekova PL, Kudrin VS (2020). The levels of monoamines and their metabolites in the brain structures of rats subjected to two- and three-month-long social isolation. Bull Exp Biol Med.

[23] Feduccia AA, Mithoefer MC (2018). MDMA-assisted psychotherapy for PTSD: are memory reconsolidation and fear extinction underlying mechanisms?. Prog Neuropsychopharmacol Biol Psychiatry.

[24] Gerlicher AMV, Tüscher O, Kalisch R (2019). L-DOPA improves extinction memory retrieval after successful fear extinction. Psychopharmacology.

[25] Lin Q, Wei W, Coelho CM, Li X, Baker-Andresen D, Dudley K (2011). The brain-specific microRNA miR-128b regulates the formation of fear-extinction memory. Nat Neurosci.

[26] Li J, Zhu L, Su H, Liu D, Yan Z, Ni T (2021). Regulation of miR-128 in the nucleus accumbens affects methamphetamine-induced behavioral sensitization by modulating proteins involved in neuroplasticity. Addict Biol.

[27] Zhou L, Yang L, Li YJ, Mei R, Yu H-L, Gong Y (2018). Wang, MicroRNA-128 protects dopamine neurons from apoptosis and upregulates the expression of excitatory amino acid transporter 4 in parkinson's disease by binding to AXIN1. Cell Physiol Biochem.

[28] Ji LL, Ye Y, Nie PY, Peng JB, Fu CH, Wang ZY (2019). Dysregulation of miR-142 results in anxiety-like behaviors following single prolonged stress. Behav Brain Res.

[29] Lee SY, Lu RB, Wang LJ, Chang CH, Lu T, Wang TY (2020). Serum miRNA as a possible biomarker in the diagnosis of bipolar II disorder. Sci Rep.

[30] Roy B, Dunbar M, Agrawal J, Allen L, Dwivedi Y (2020). Amygdala-based altered mirnome and epigenetic contribution of miR-128-3p in conferring susceptibility to depression-like behavior via wnt signaling. Int J Neuropsychopharmacol.

[31] Lehner M, Wisłowska-Stanek A, Taracha E, Maciejak P, Szyndler J, Skórzewska A (2010). The effects of midazolam and D-cycloserine on the release of glutamate and GABA in the basolateral amygdala of low and high anxiety rats during extinction trial of a conditioned fear test. Neurobiol Learn Mem.

[32] Hamed A, Szyndler J, Taracha E, Turzyńska D, Sobolewska A, Lehner M (2015). κ-opioid receptor as a key mediator in the regulation of appetitive 50-kHz ultrasonic vocalizations. Psychopharmacology.

[33] Paxinos G , Watson CH. The rat brain in stereotaxic coordinates. San Diego: Academic Press,1998.

[34] Kołosowska K, Gawryluk A, Wisłowska-Stanek A, Liguz-Lęcznar M, Hetmańczyk K, Ługowska A (2019). Stress changes amphetamine response, D2 receptor expression and epigenetic regulation in low-anxiety rats. Prog Neuropsychopharmacol Biol Psychiatry.

[35] Nunes Mamede Rosa ML, Nobre MJ, Ribeiro Oliveira A, Brandão ML (2005). Isolation-induced changes in ultrasonic vocalization, fear-potentiated startle and prepulse inhibition in rats. Neuropsychobiology.

[36] Pêgo JM, Sousa JC, Almeida OF, Sousa N (2010). Stress and the neuroendocrinology of anxiety disorders. Curr Top Behav Neurosci.

[37] Pinna G (2019). Animal models of PTSD: the socially isolated mouse and the biomarker role of allopregnanolone. Front Behav Neurosci.

[38] Butler TR, Karkhanis AN, Jones SR, Weiner JL (2016). Adolescent social isolation as a model of heightened vulnerability to comorbid alcoholism and anxiety disorders. Alcohol Clin Exp Res.

[39] Cuenya L, Fosacheca S, Mustaca A, Kamenetzky G (2012). Effects of isolation in adulthood on frustration and anxiety. Behav Processes.

[40] Locci A, Pinna G (2019). Social isolation as a promising animal model of PTSD comorbid suicide: neurosteroids and cannabinoids as possible treatment options. Prog Neuropsychopharmacol Biol Psychiatry.

[41] Haj-Mirzaian A, Nikbakhsh R, Ramezanzadeh K, Rezaee M, Amini-Khoei H, Haj-Mirzaian A (2019). Involvement of opioid system in behavioral despair induced by social isolation stress in mice. Biomed Pharmacother.

[42] LeDoux JE, Iwata J, Cicchetti P, Reis DJ (1988). Different projections of the central amygdaloid nucleus mediate autonomic and behavioral correlates of conditioned fear. J Neurosci.

[43] Nazari-Serenjeh F, Zarrabian S, Azizbeigi R, Haghparast A (2021). Effects of dopamine D1- and D2-like receptors in the CA1 region of the hippocampus on expression and extinction of morphine-induced conditioned place preference in rats. Behav Brain Res.

[44] Adlakha YK, Saini N (2014). Brain microRNAs and insights into biological functions and therapeutic potential of brain enriched miRNA-128. Mol Cancer.

