# Supplementary figures and images for: The effects of the recurrent social isolation stress on fear extinction and dopamine D2 receptors in the amygdala and the hippocampus
Source: Pharmacol Rep. 2022 Nov 17;75(1):119–27. doi: 10.1007/s43440-022-00430-8 (PMC9889440; doi:10.1007/s43440-022-00430-8)

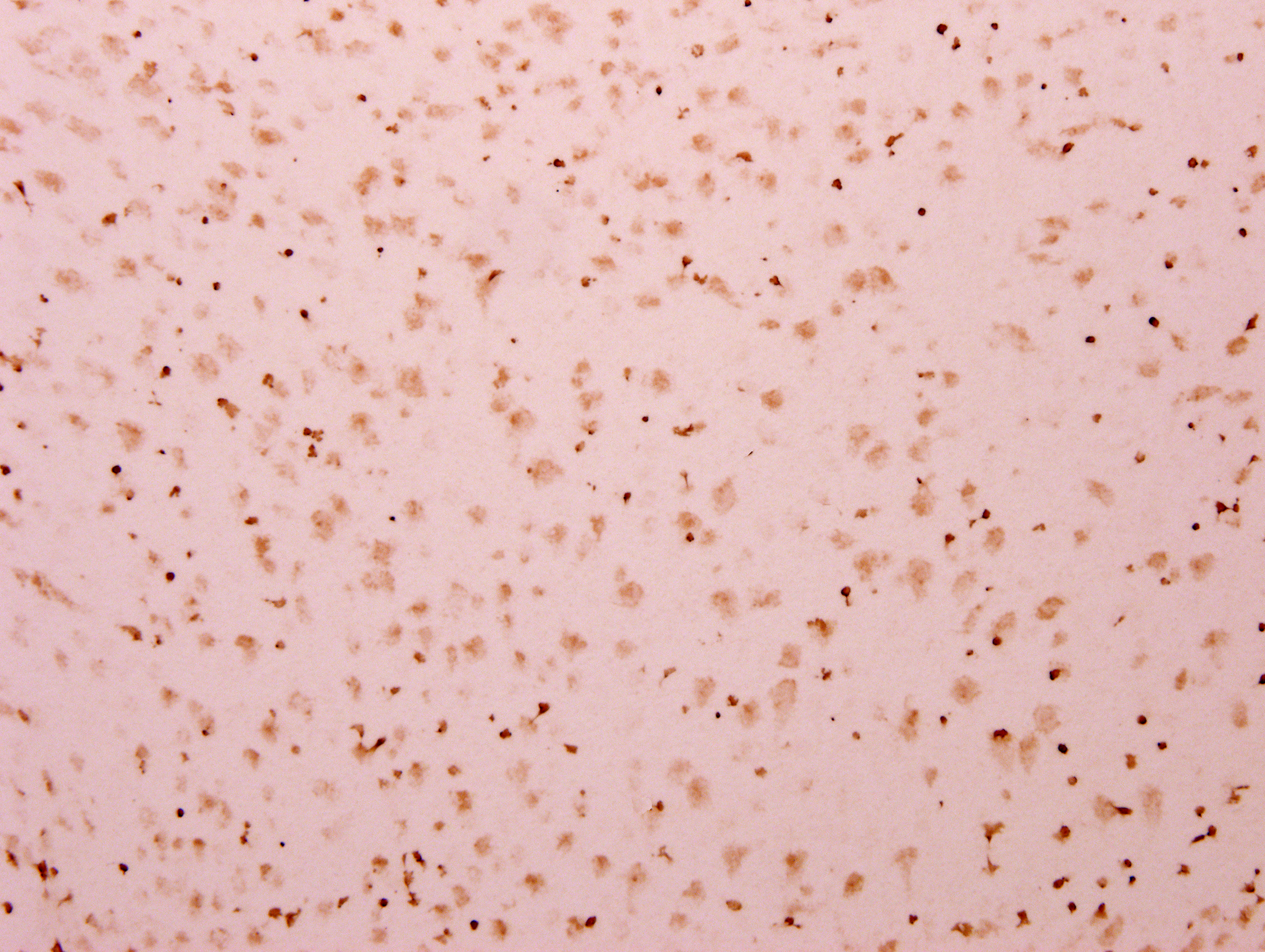

Supplement: Supplementary file 1 — Supplementary file1 (TIF 29680 KB) [file 43440_2022_430_MOESM1_ESM.tif]

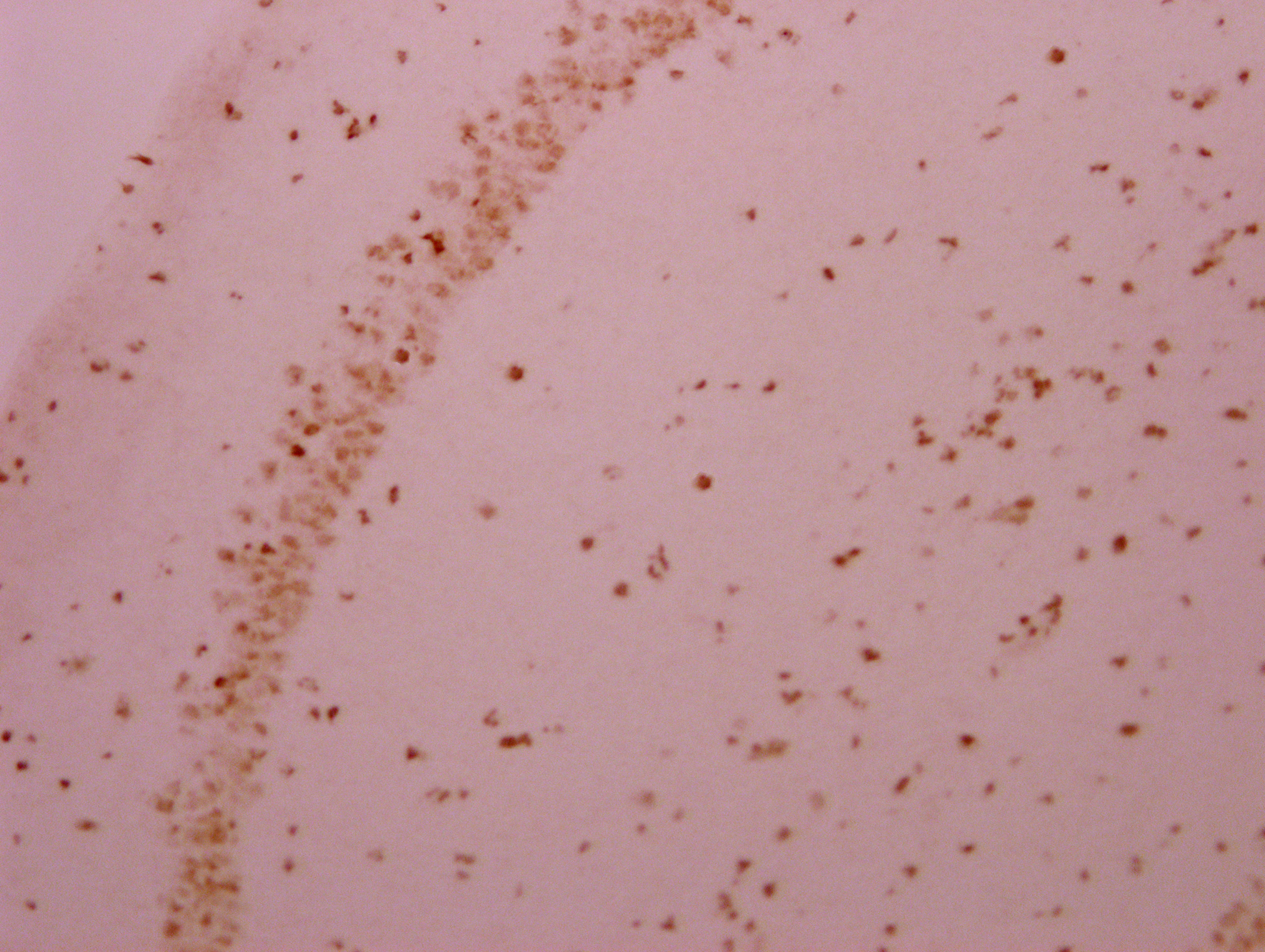

Supplement: Supplementary file 2 — Supplementary file2 (TIF 28033 KB) [file 43440_2022_430_MOESM2_ESM.tif]

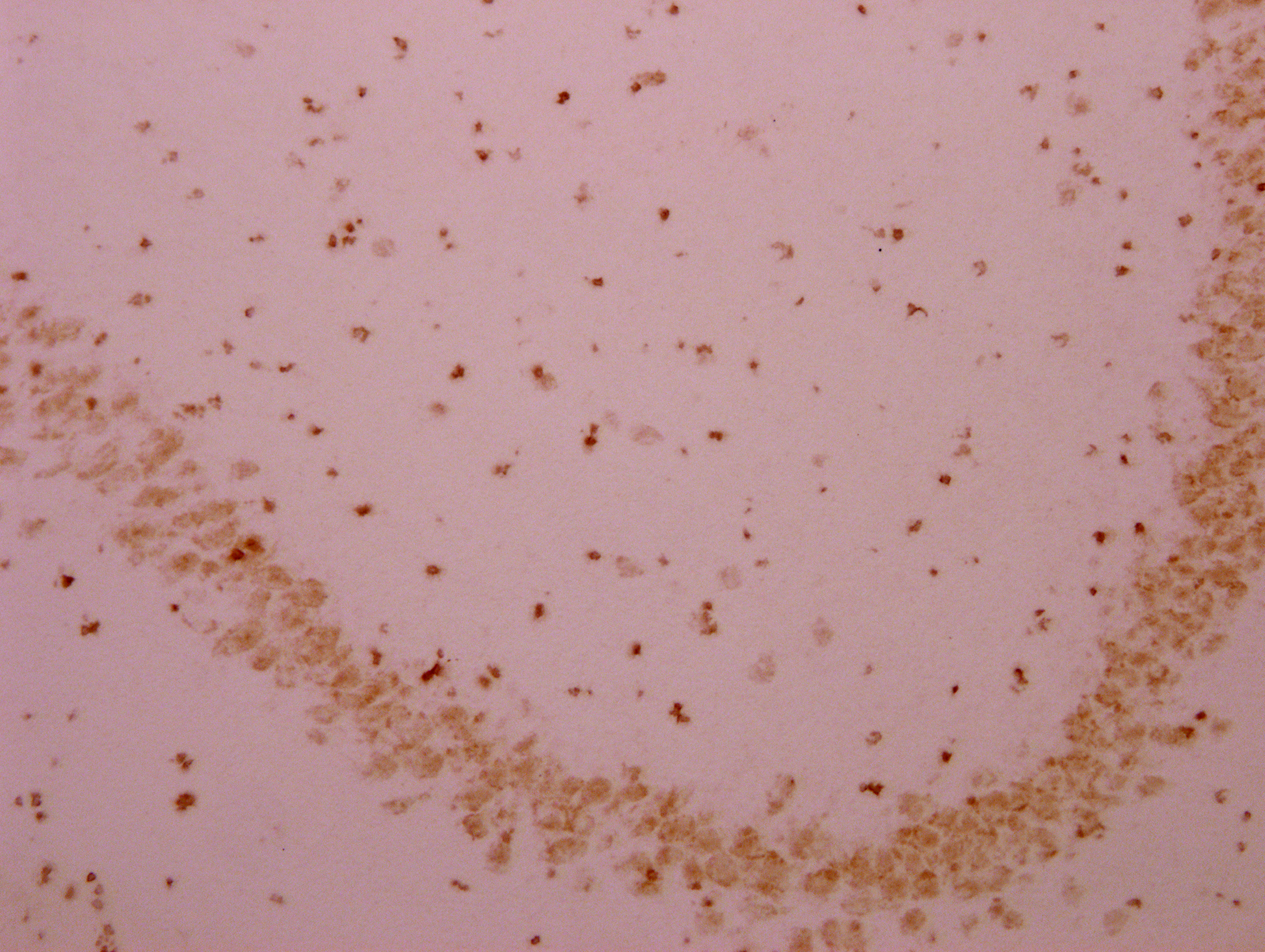

Supplement: Supplementary file 3 — Supplementary file3 (TIF 27980 KB) [file 43440_2022_430_MOESM3_ESM.tif]

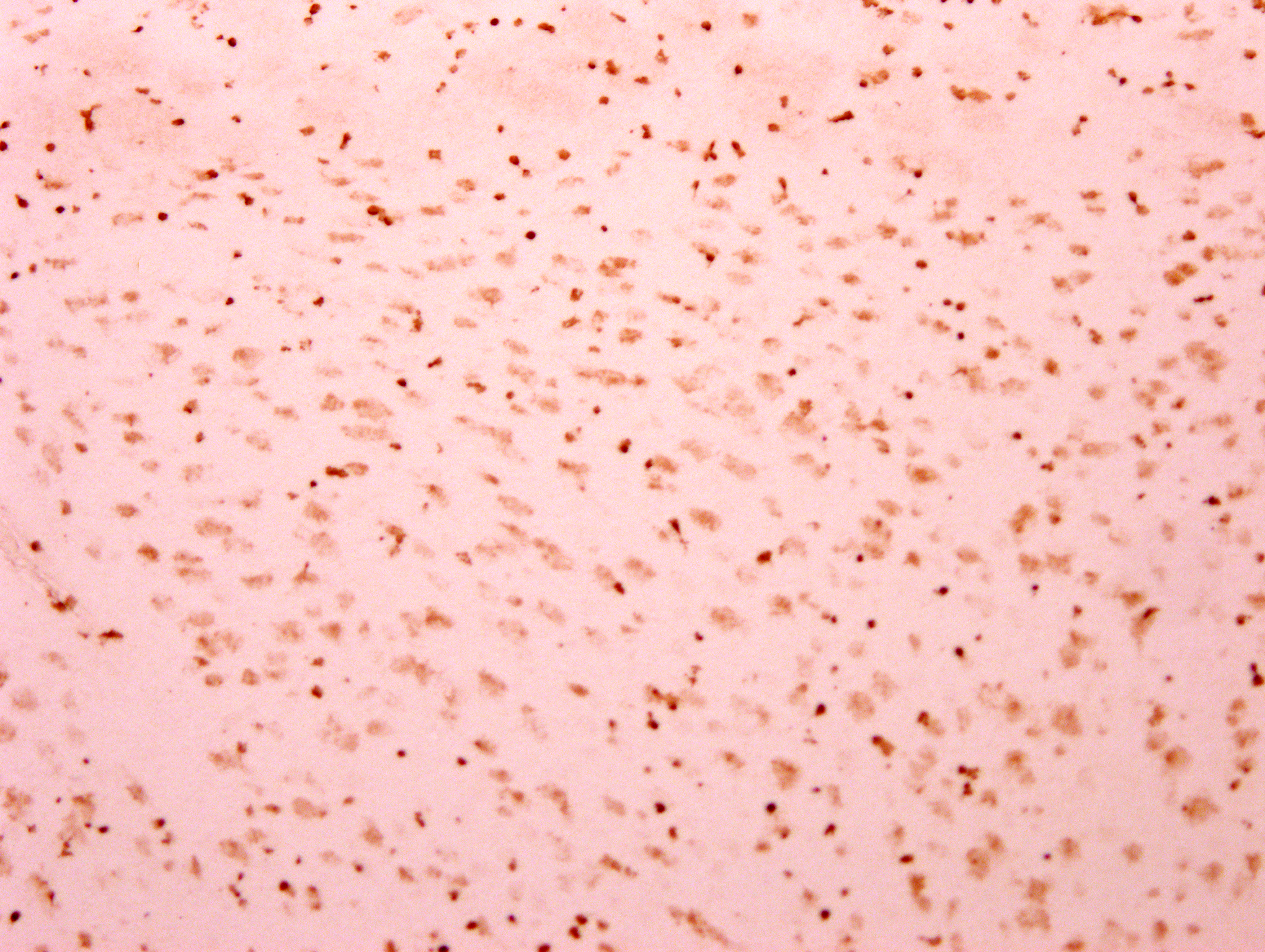

Supplement: Supplementary file 4 — Supplementary file4 (TIF 26376 KB) [file 43440_2022_430_MOESM4_ESM.tif]

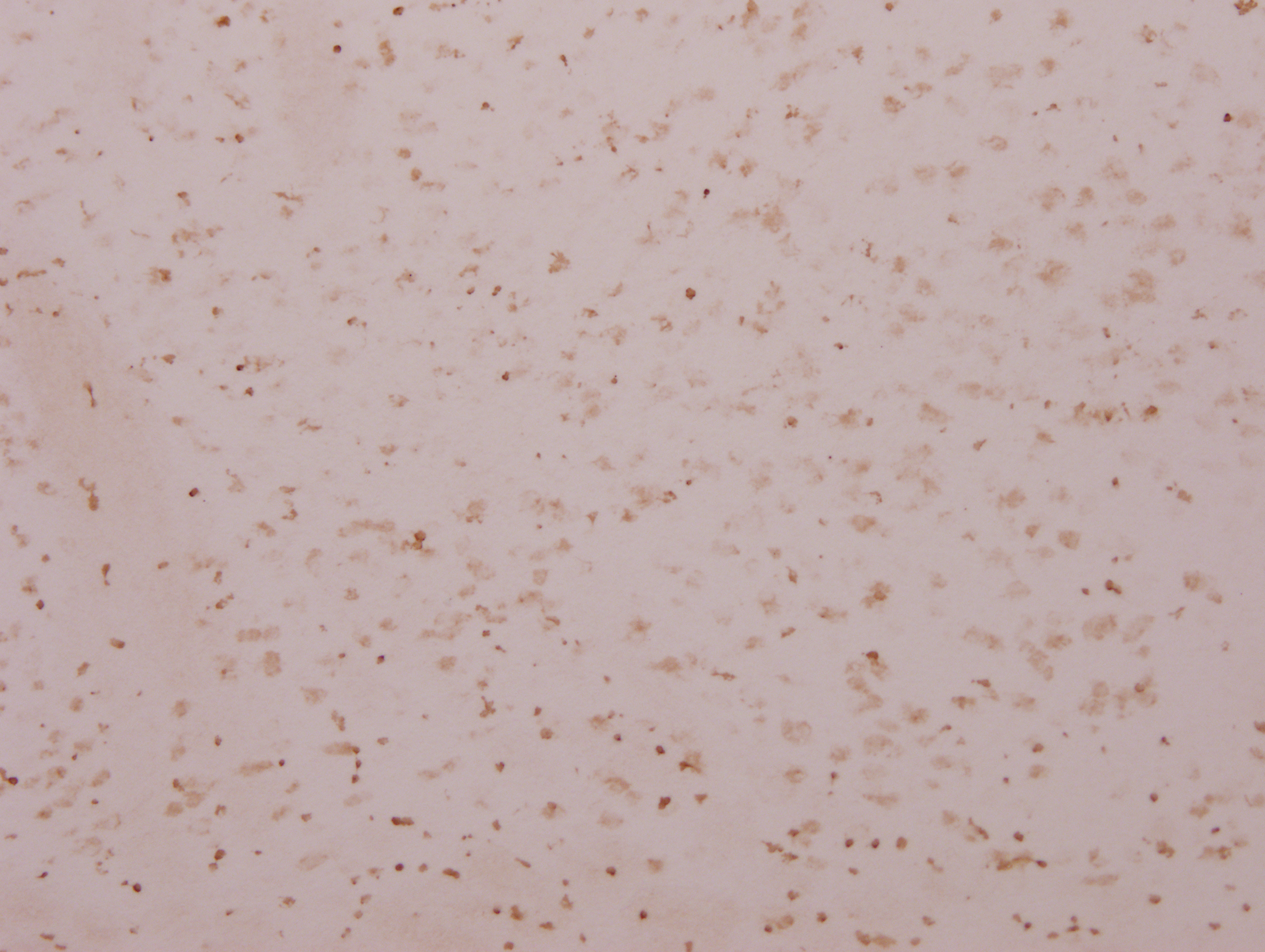

Supplement: Supplementary file 5 — Supplementary file5 (TIF 17919 KB) [file 43440_2022_430_MOESM5_ESM.tif]

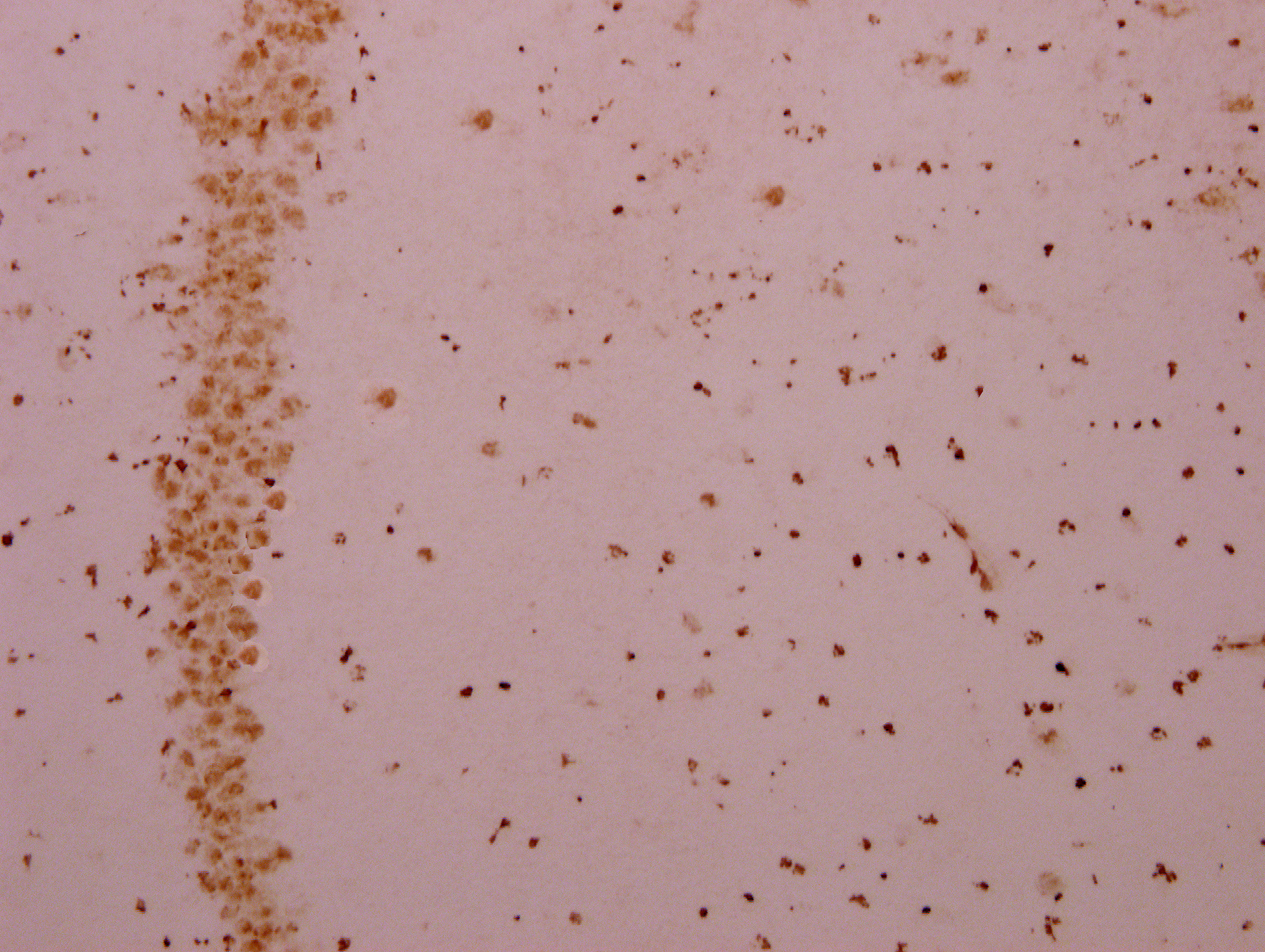

Supplement: Supplementary file 6 — Supplementary file6 (TIF 40613 KB) [file 43440_2022_430_MOESM6_ESM.tif]

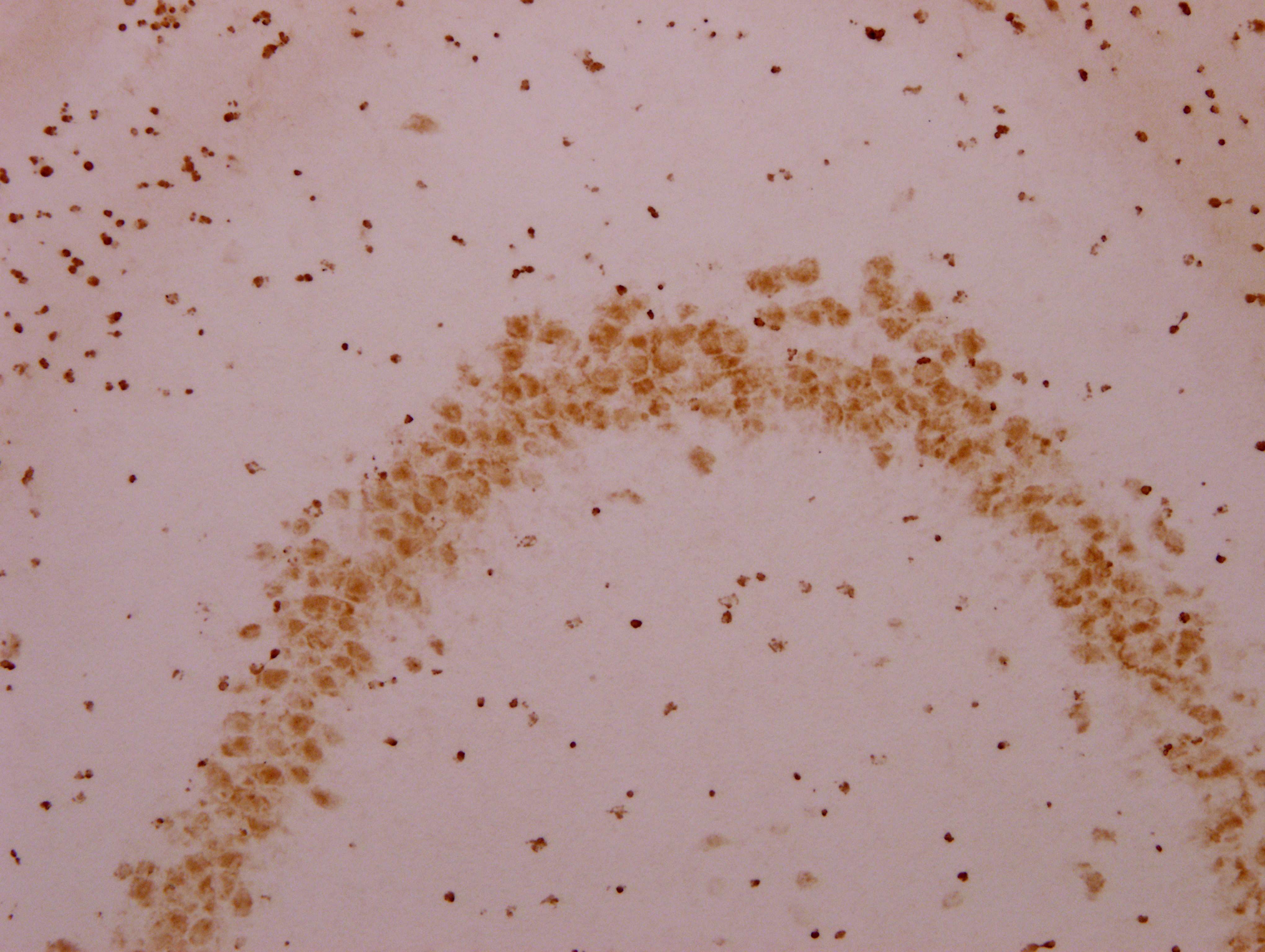

Supplement: Supplementary file 7 — Supplementary file7 (TIF 25097 KB) [file 43440_2022_430_MOESM7_ESM.tif]

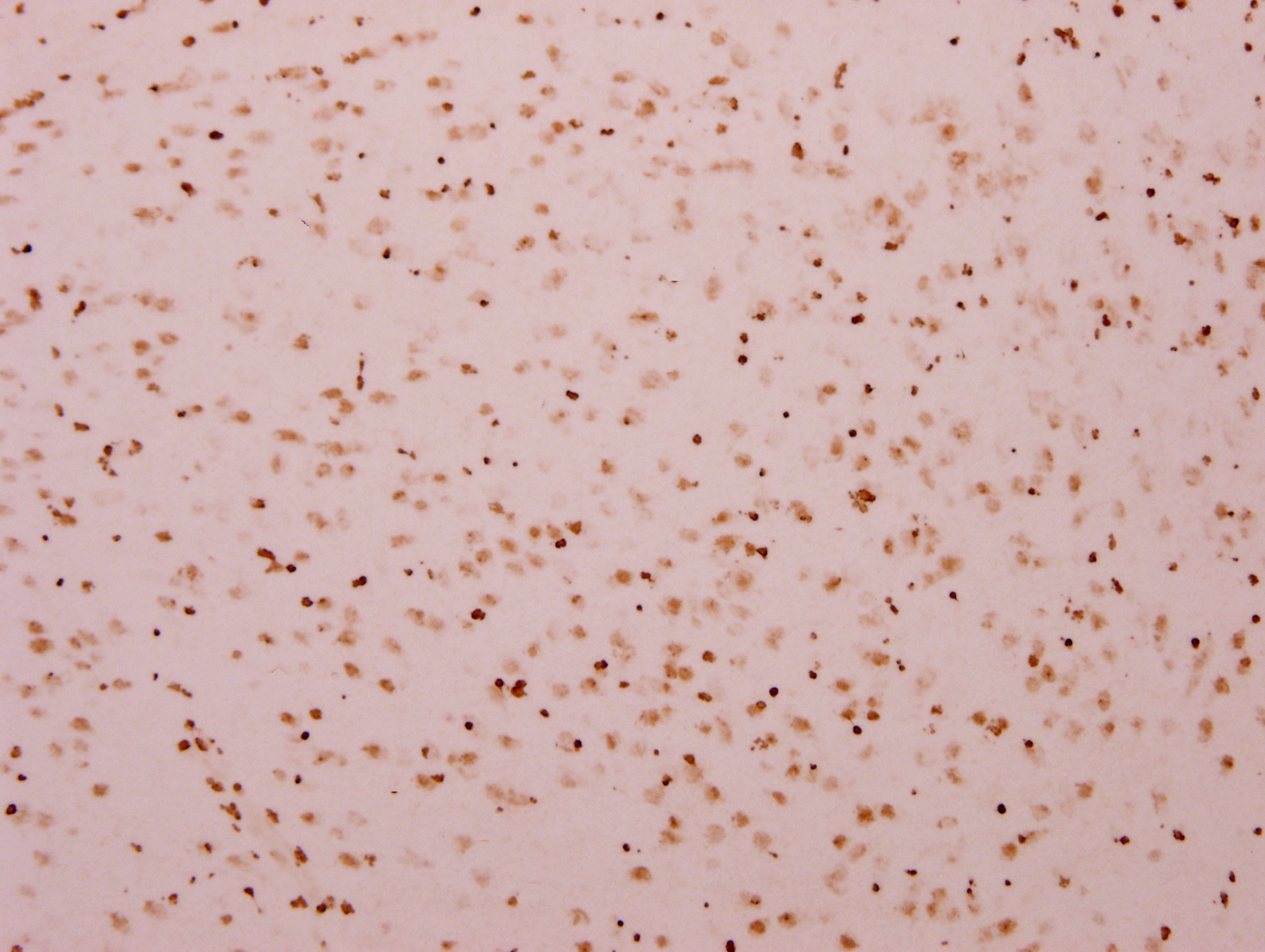

Supplement: Supplementary file 8 — Supplementary file8 (TIF 24081 KB) [file 43440_2022_430_MOESM8_ESM.tif]

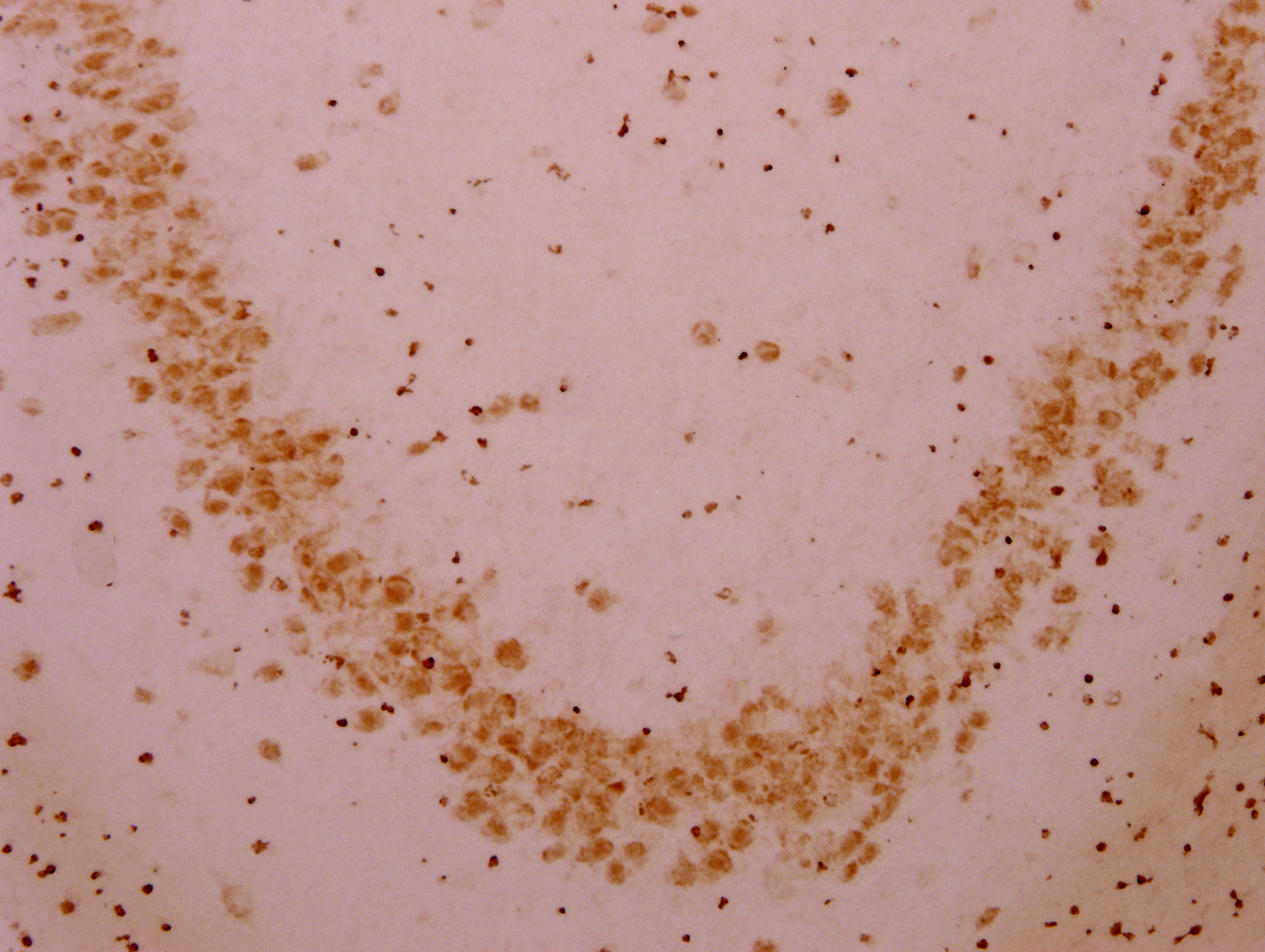

Supplement: Supplementary file 9 — Supplementary file9 (TIF 29241 KB) [file 43440_2022_430_MOESM9_ESM.tif]

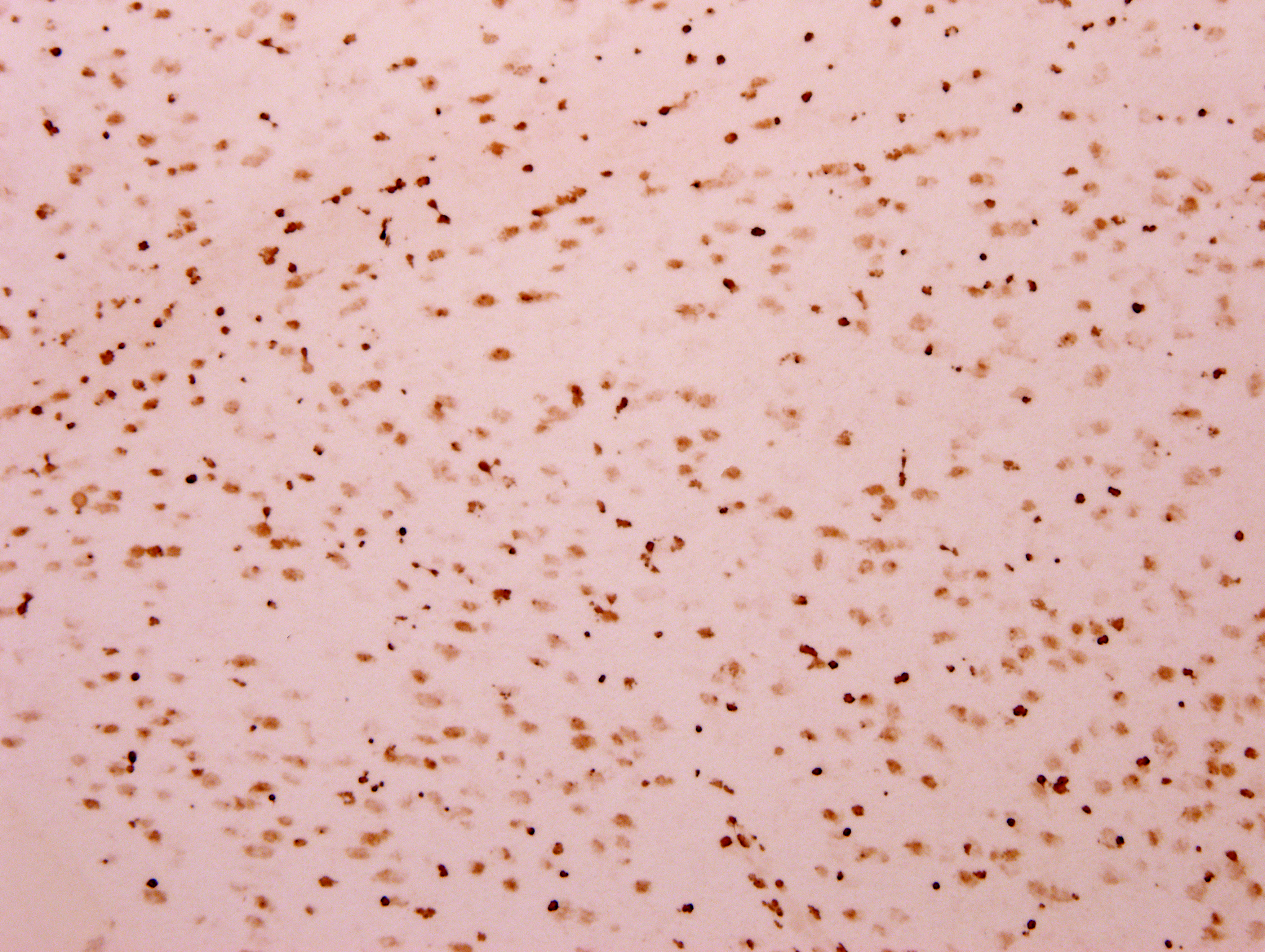

Supplement: Supplementary file 10 — Supplementary file10 (TIF 25981 KB) [file 43440_2022_430_MOESM10_ESM.tif]

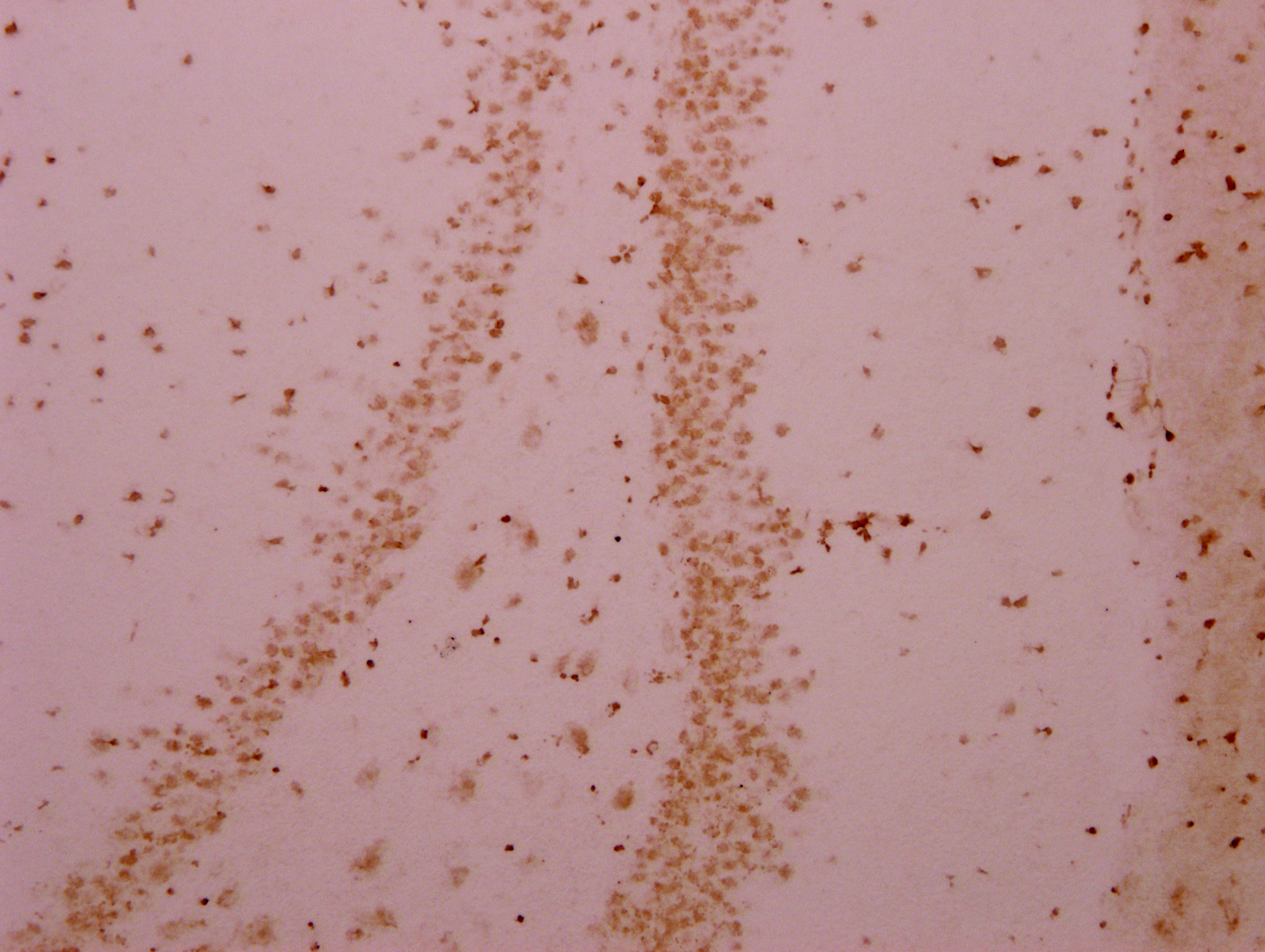

Supplement: Supplementary file 11 — Supplementary file11 (TIF 27647 KB) [file 43440_2022_430_MOESM11_ESM.tif]

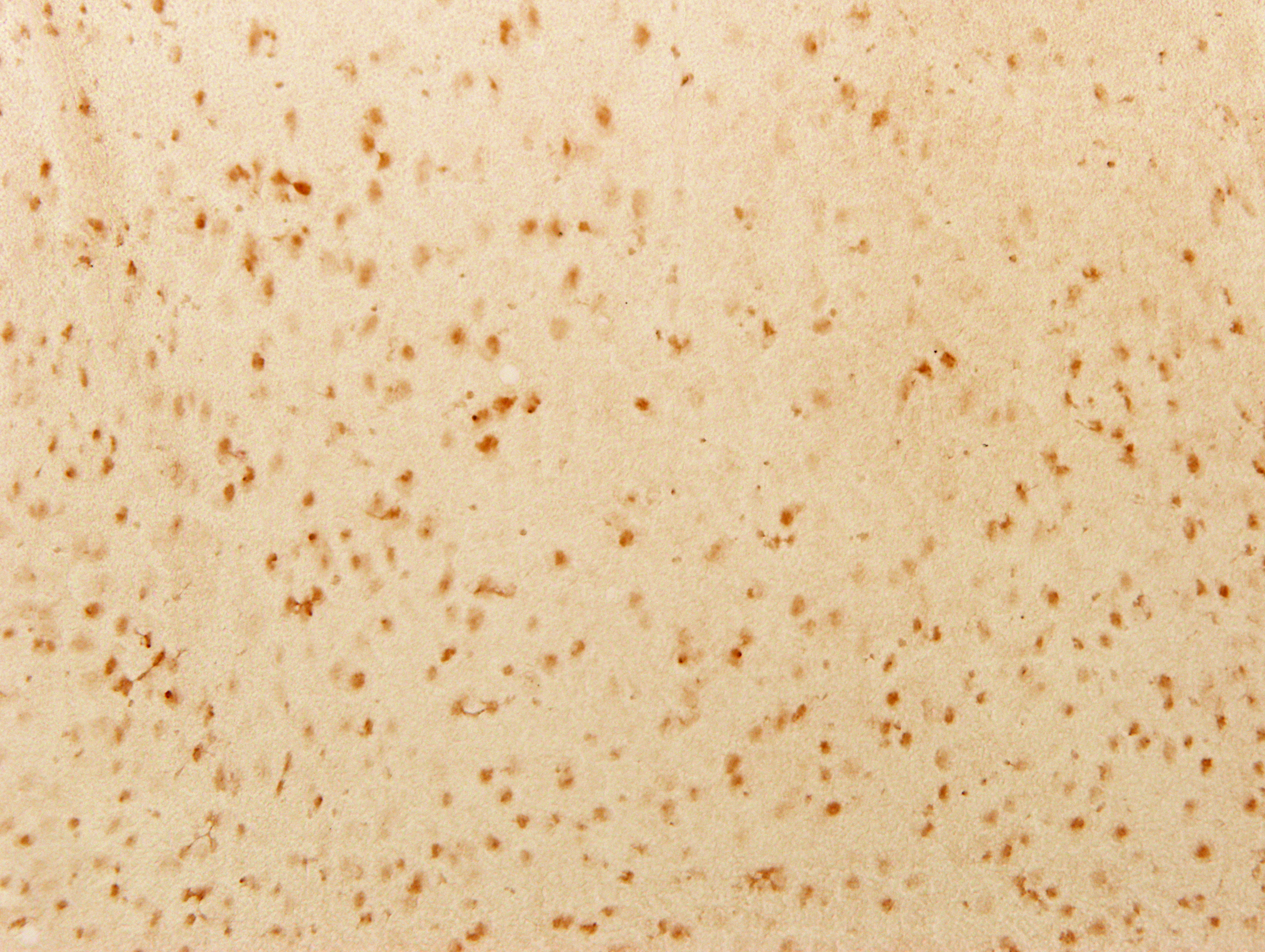

Supplement: Supplementary file 12 — Supplementary file12 (TIF 26288 KB) [file 43440_2022_430_MOESM12_ESM.tif]

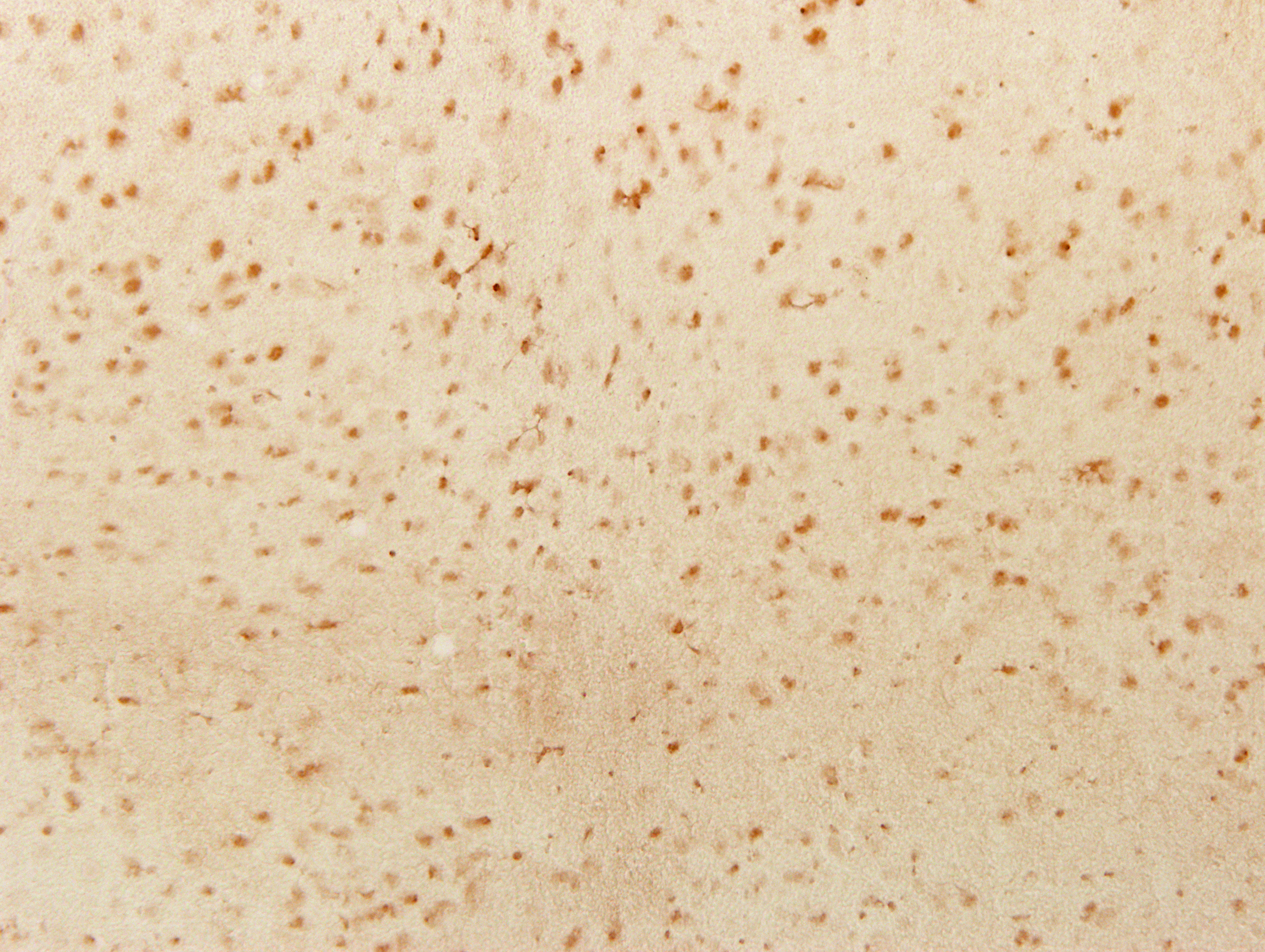

Supplement: Supplementary file 13 — Supplementary file13 (TIF 26101 KB) [file 43440_2022_430_MOESM13_ESM.tif]

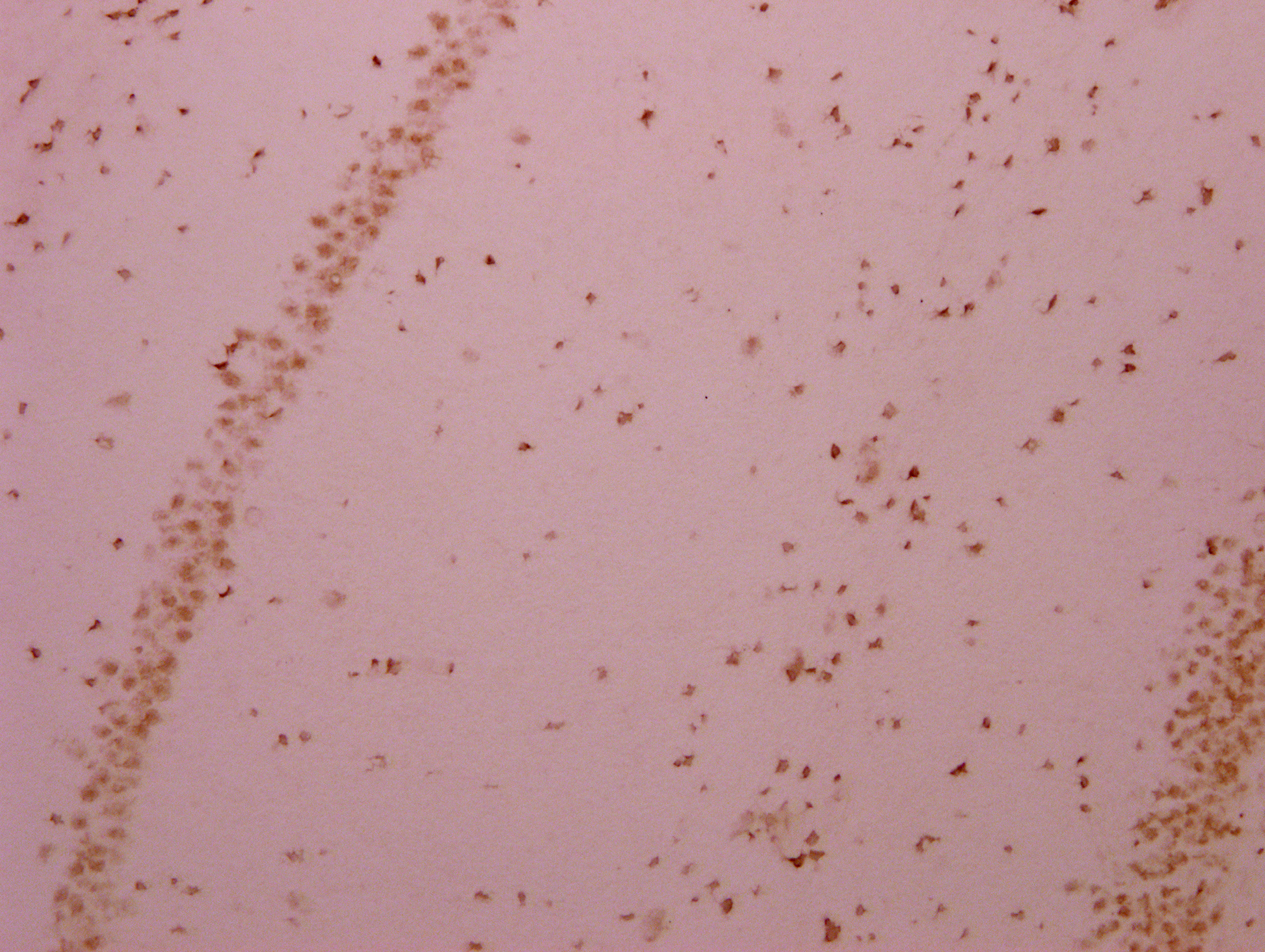

Supplement: Supplementary file 14 — Supplementary file14 (TIF 28155 KB) [file 43440_2022_430_MOESM14_ESM.tif]

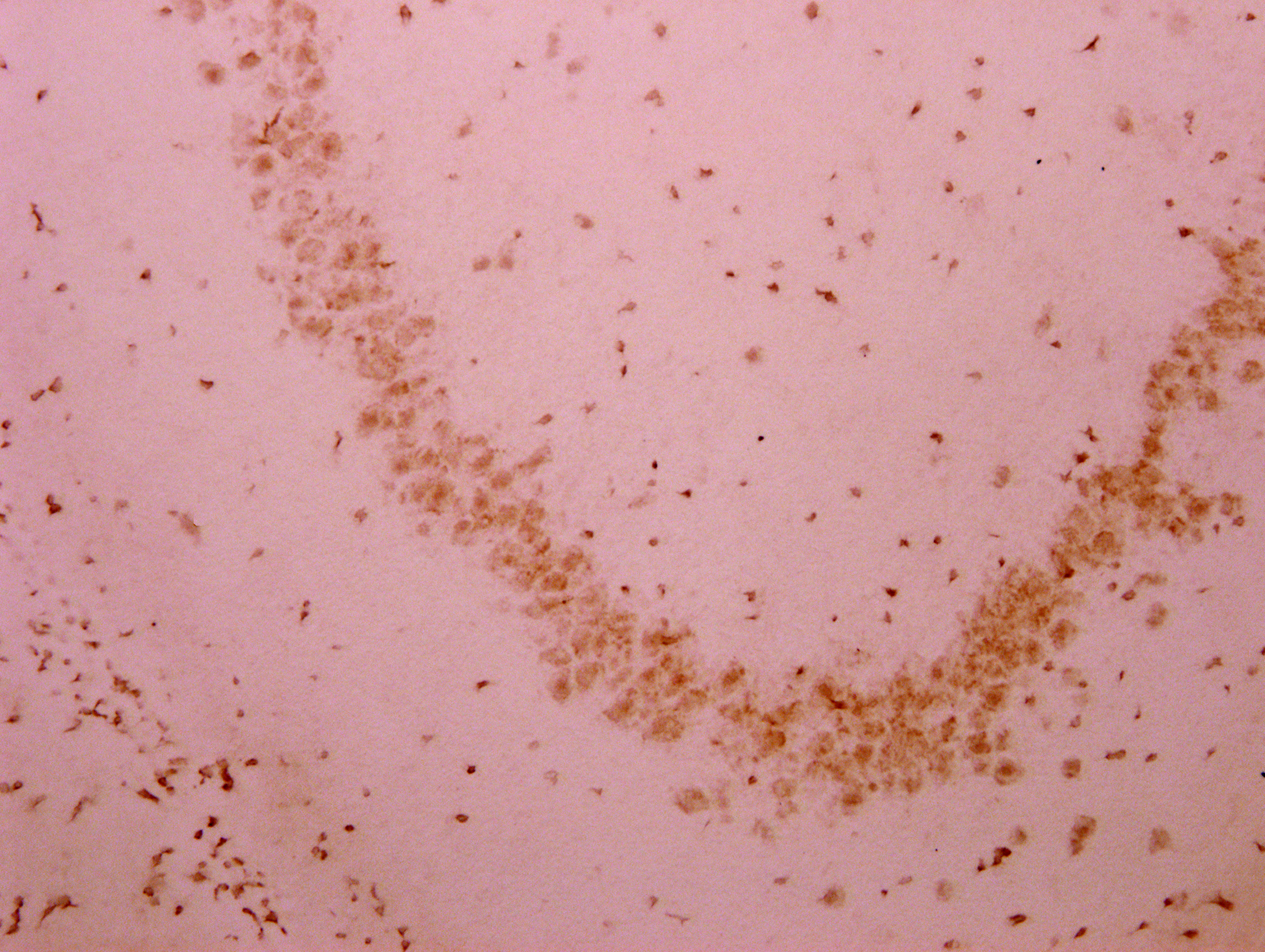

Supplement: Supplementary file 15 — Supplementary file15 (TIF 27368 KB) [file 43440_2022_430_MOESM15_ESM.tif]

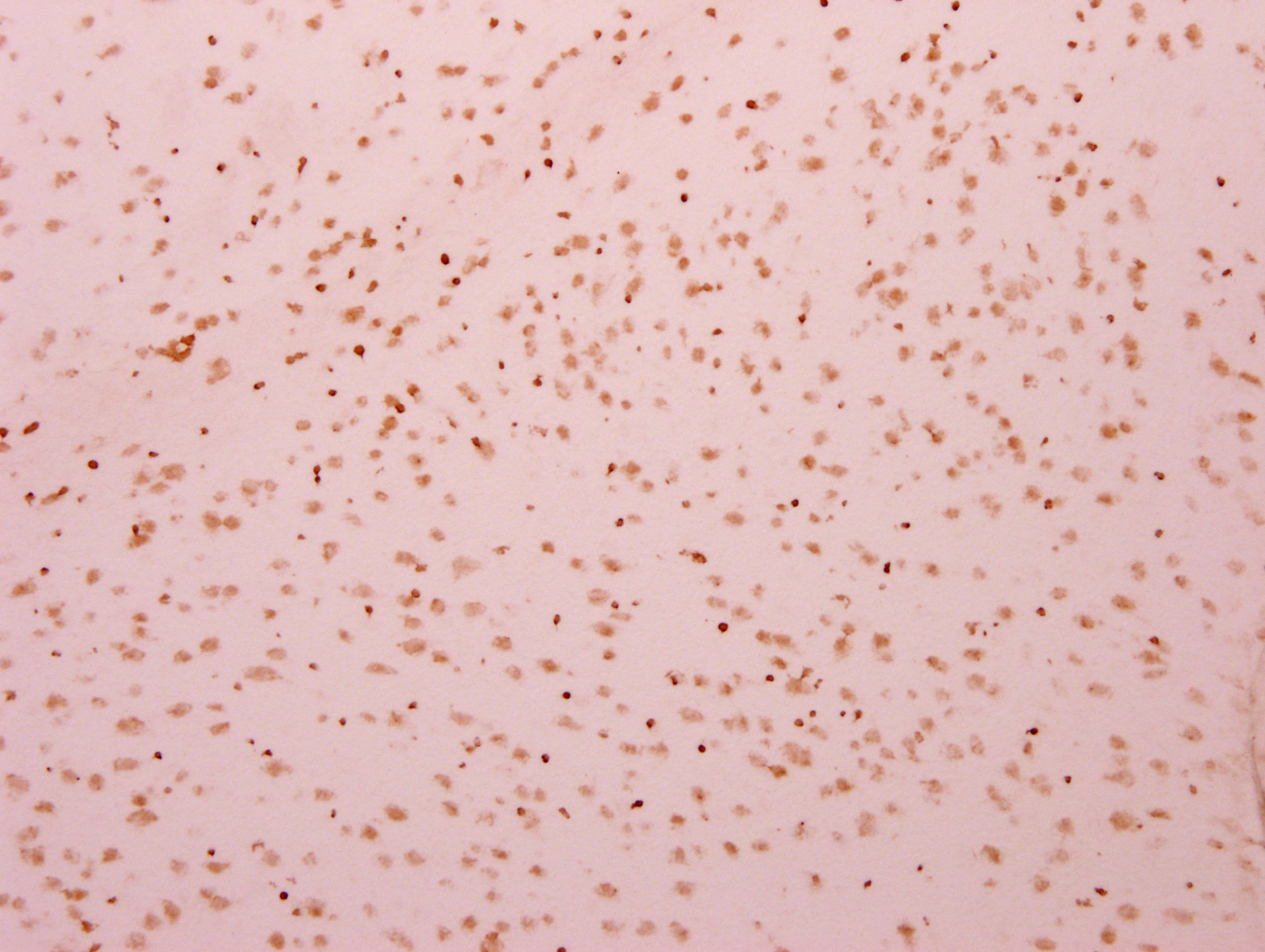

Supplement: Supplementary file 16 — Supplementary file16 (TIF 29186 KB) [file 43440_2022_430_MOESM16_ESM.tif]

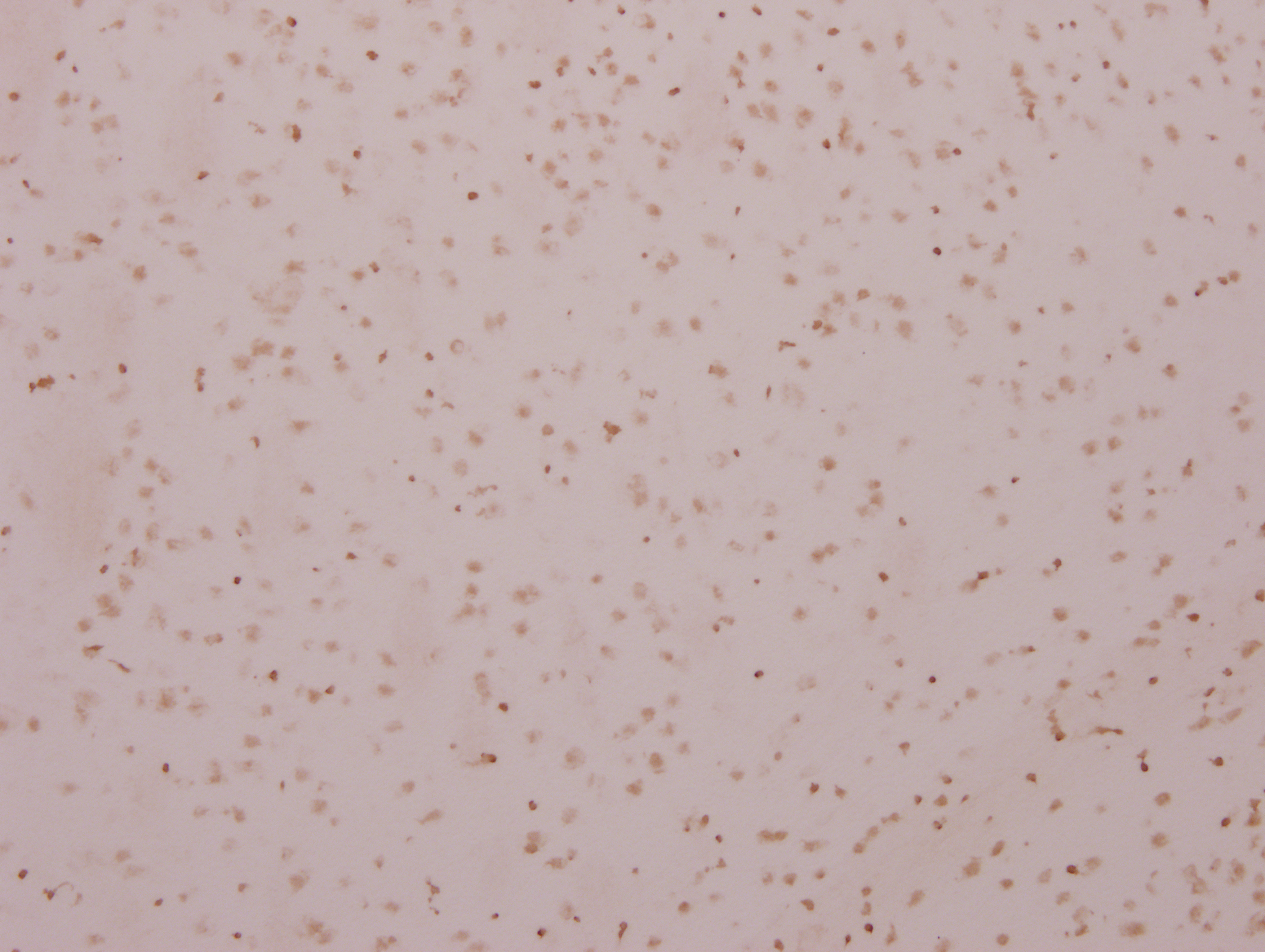

Supplement: Supplementary file 17 — Supplementary file17 (TIF 17845 KB) [file 43440_2022_430_MOESM17_ESM.tif]

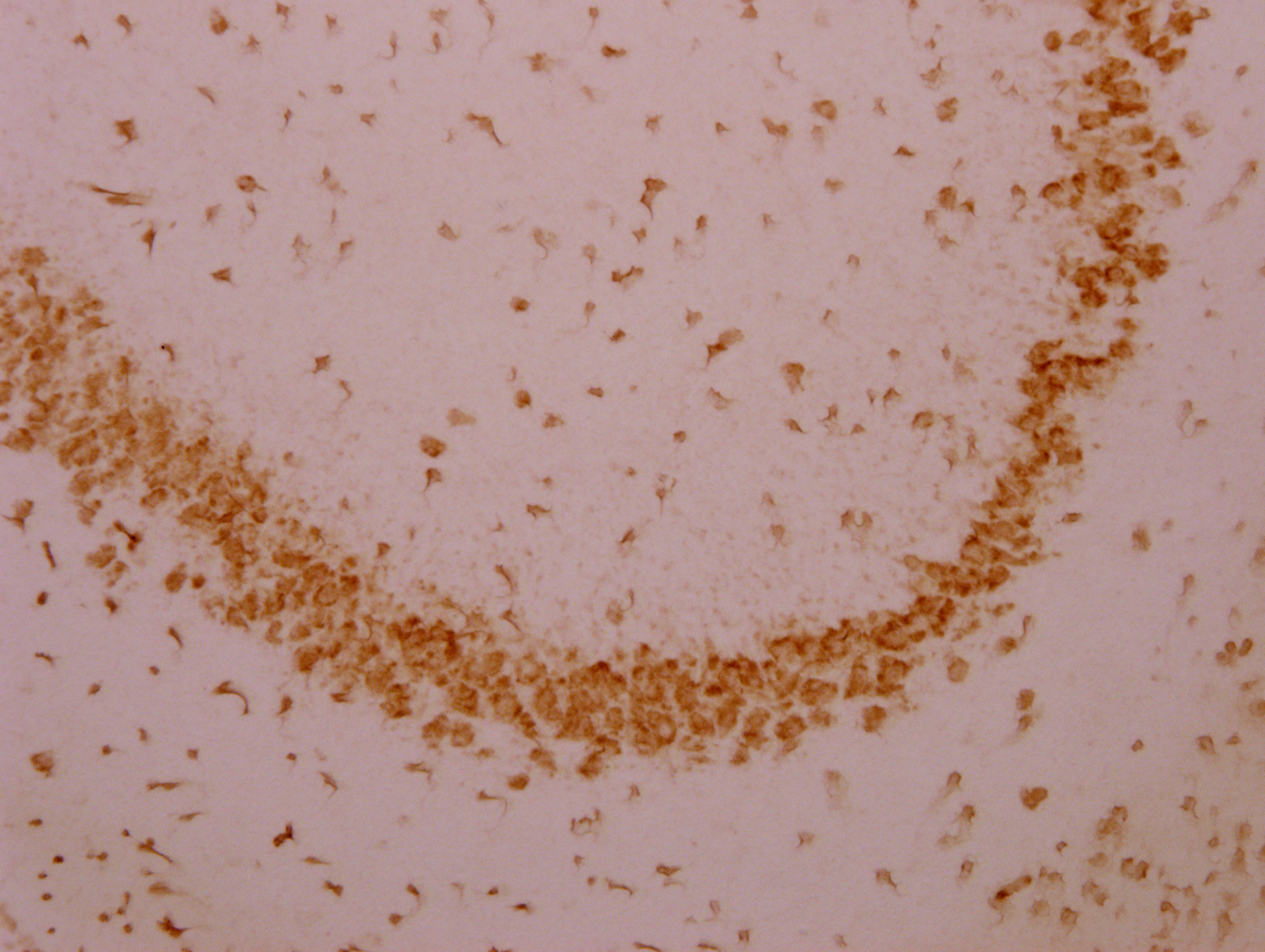

Supplement: Supplementary file 18 — Supplementary file18 (TIF 25502 KB) [file 43440_2022_430_MOESM18_ESM.tif]

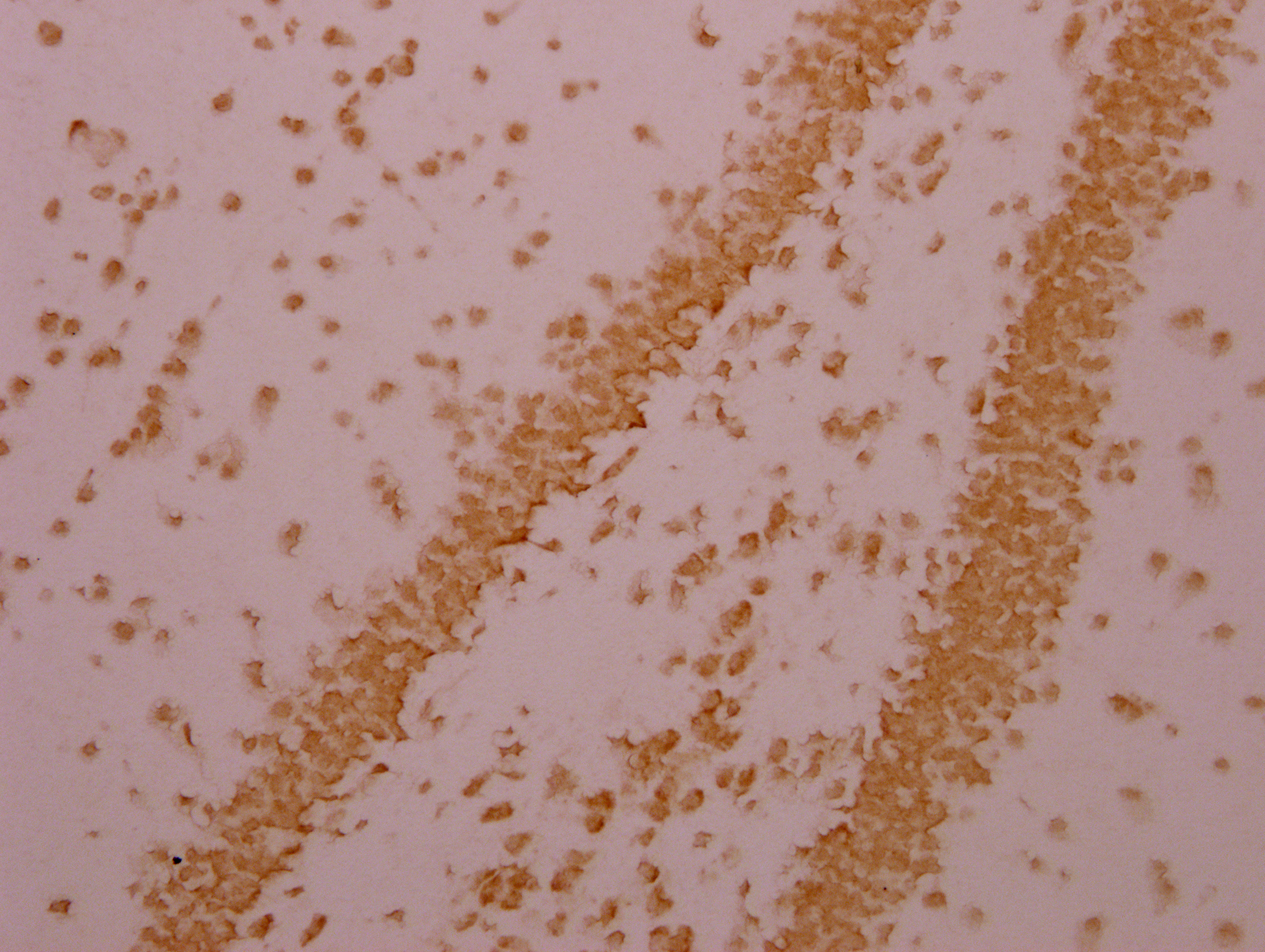

Supplement: Supplementary file 19 — Supplementary file19 (TIF 26149 KB) [file 43440_2022_430_MOESM19_ESM.tif]

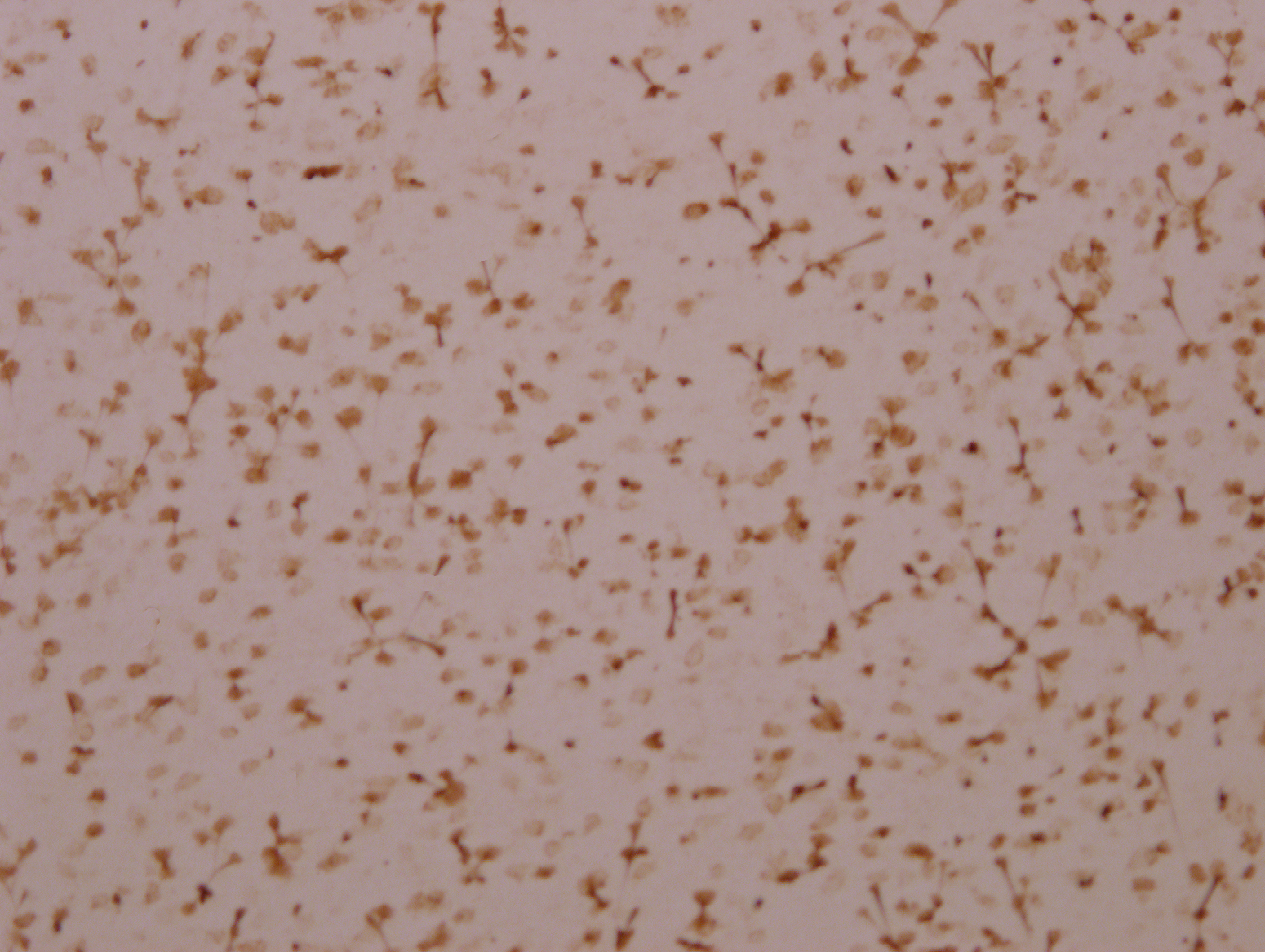

Supplement: Supplementary file 20 — Supplementary file20 (TIF 20142 KB) [file 43440_2022_430_MOESM20_ESM.tif]

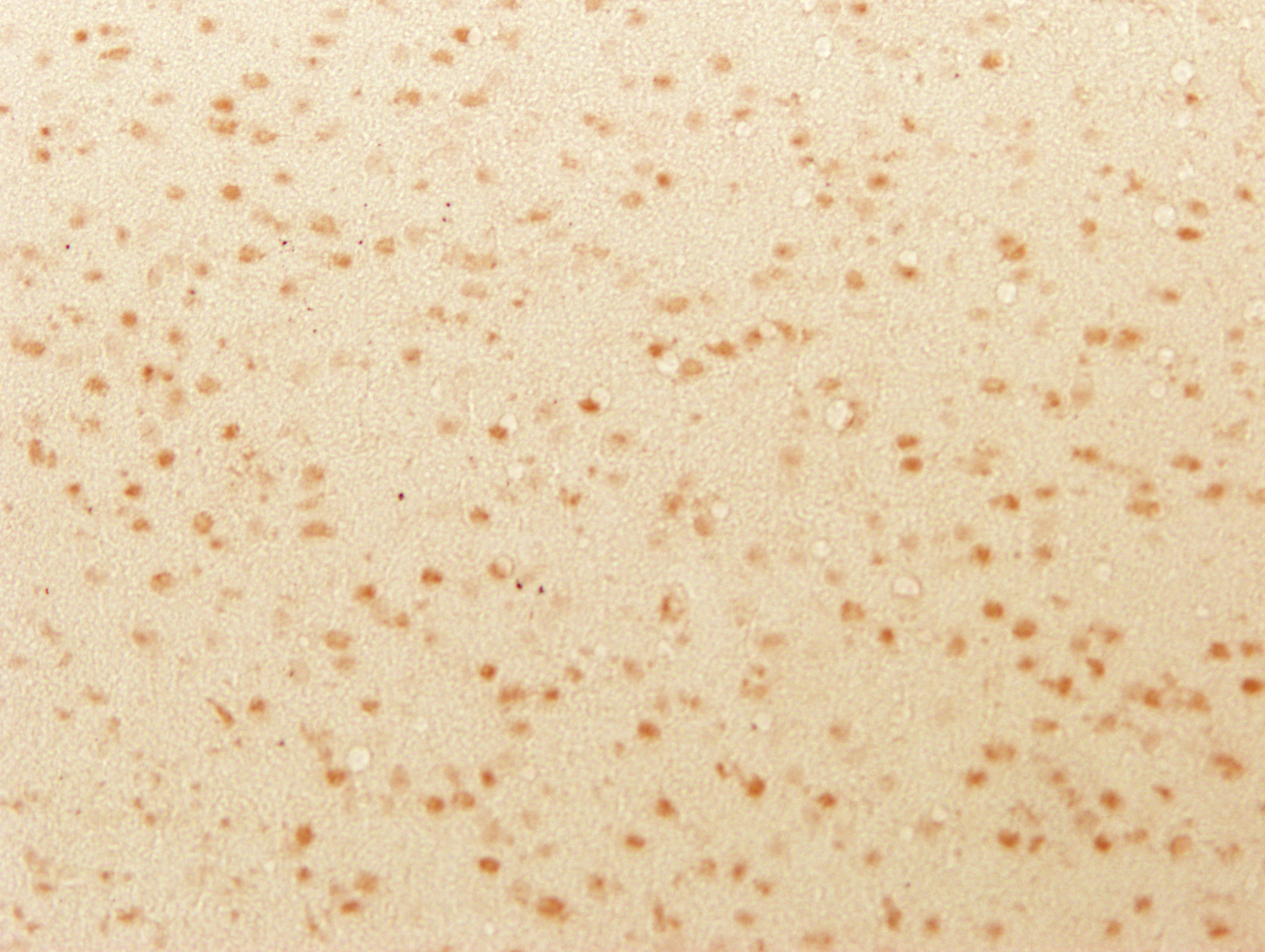

Supplement: Supplementary file 21 — Supplementary file21 (TIF 25877 KB) [file 43440_2022_430_MOESM21_ESM.tif]

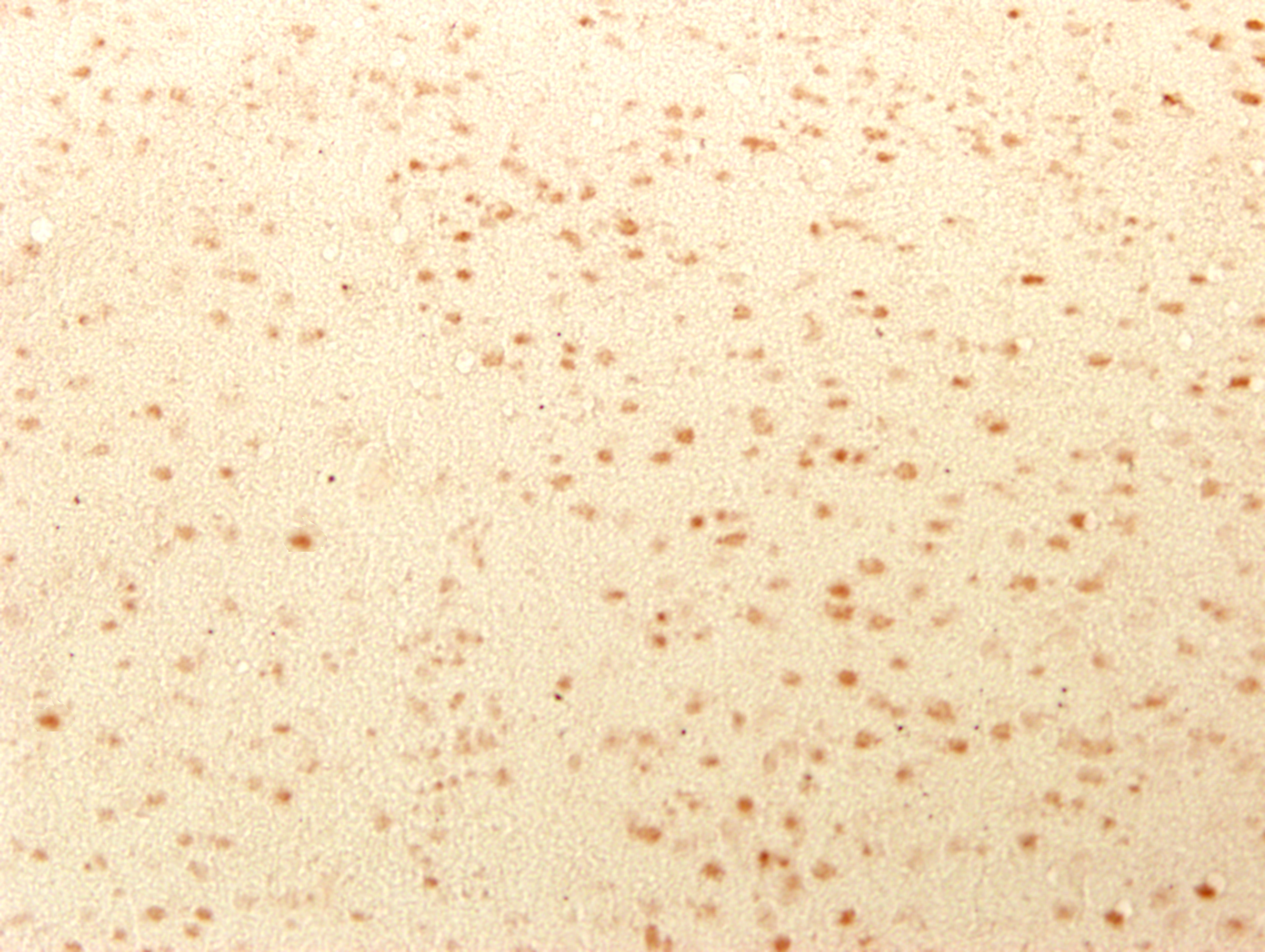

Supplement: Supplementary file 22 — Supplementary file22 (TIF 13394 KB) [file 43440_2022_430_MOESM22_ESM.tif]

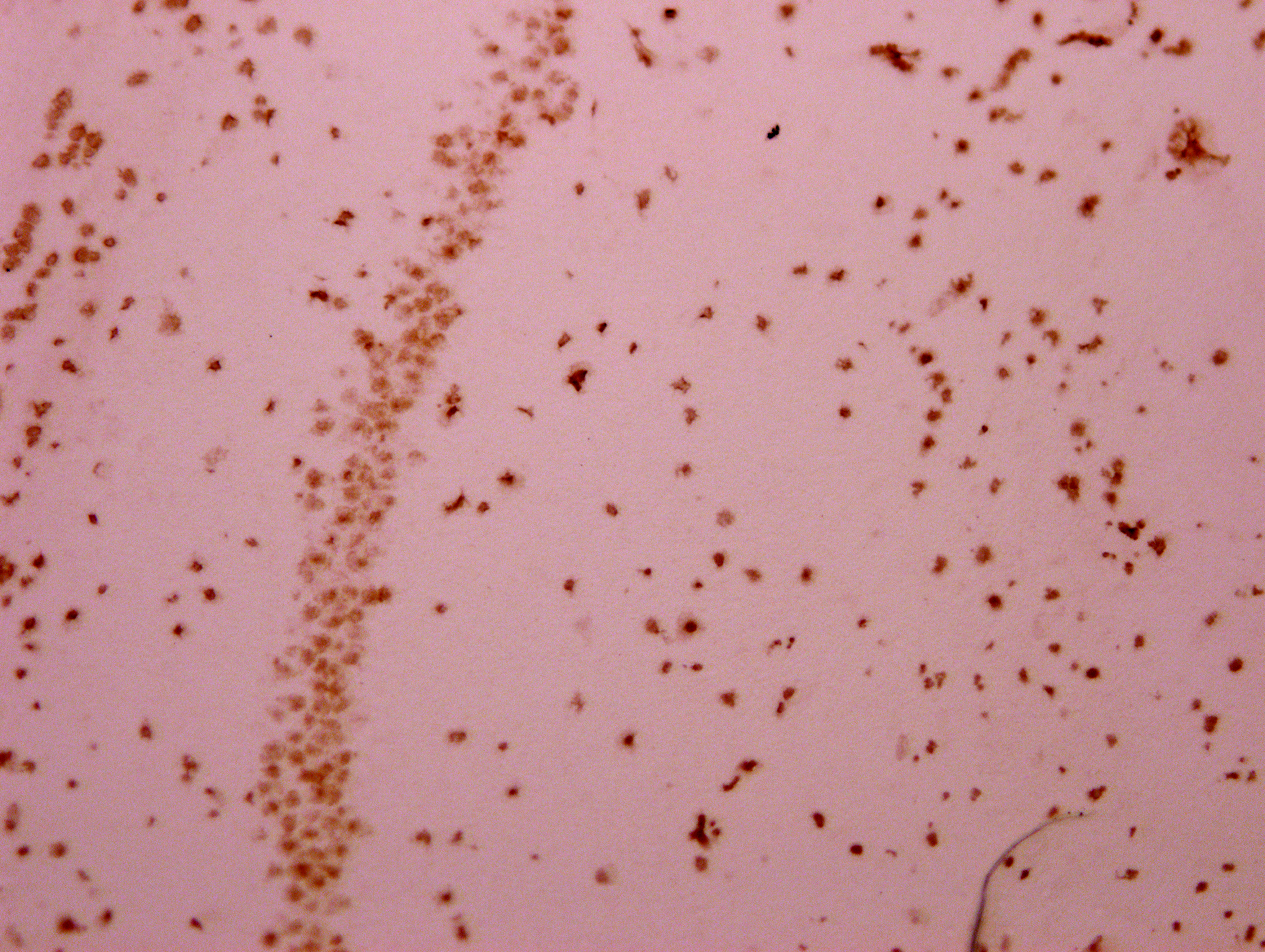

Supplement: Supplementary file 23 — Supplementary file23 (TIF 26950 KB) [file 43440_2022_430_MOESM23_ESM.tif]

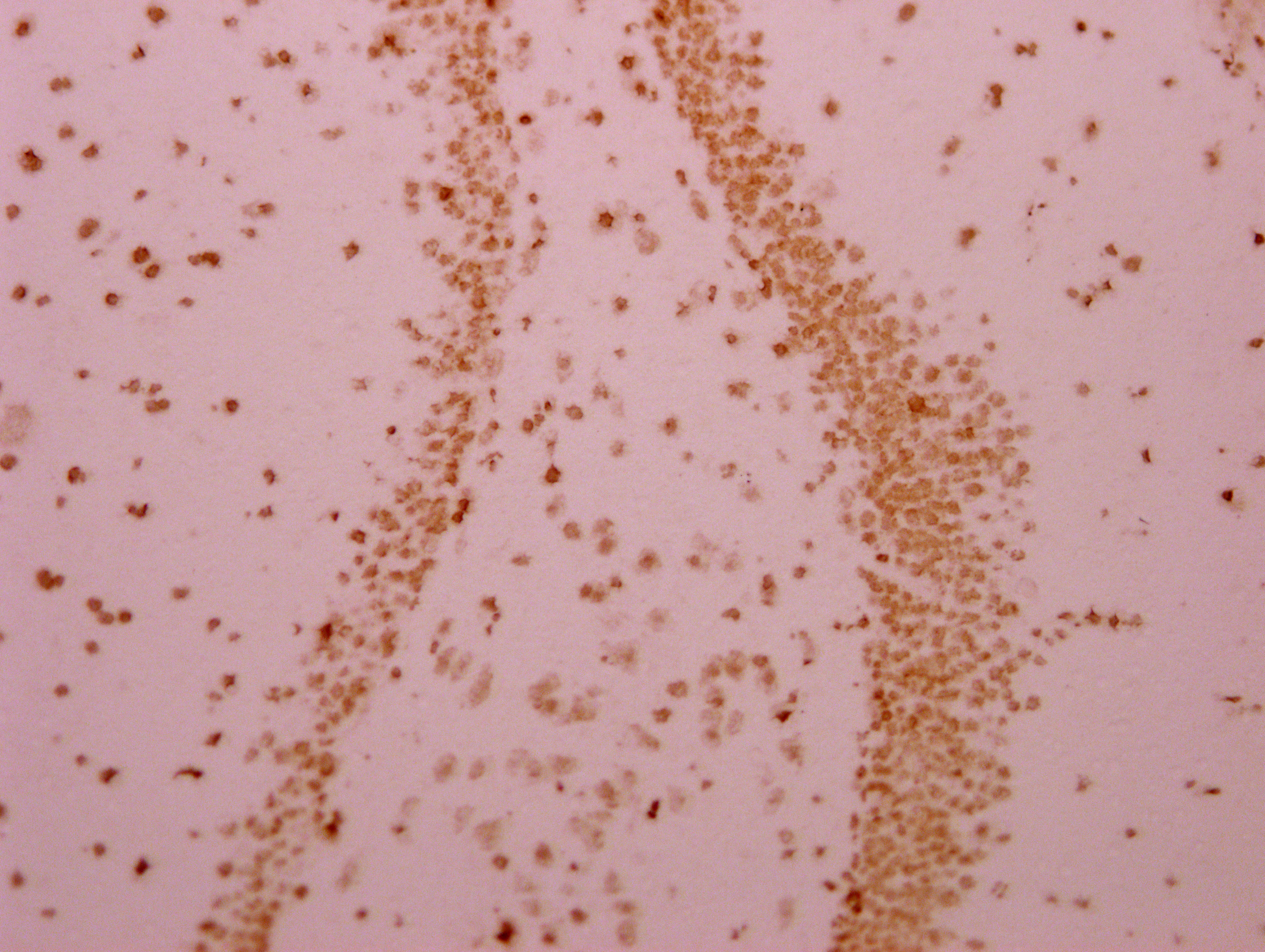

Supplement: Supplementary file 24 — Supplementary file24 (TIF 31775 KB) [file 43440_2022_430_MOESM24_ESM.tif]

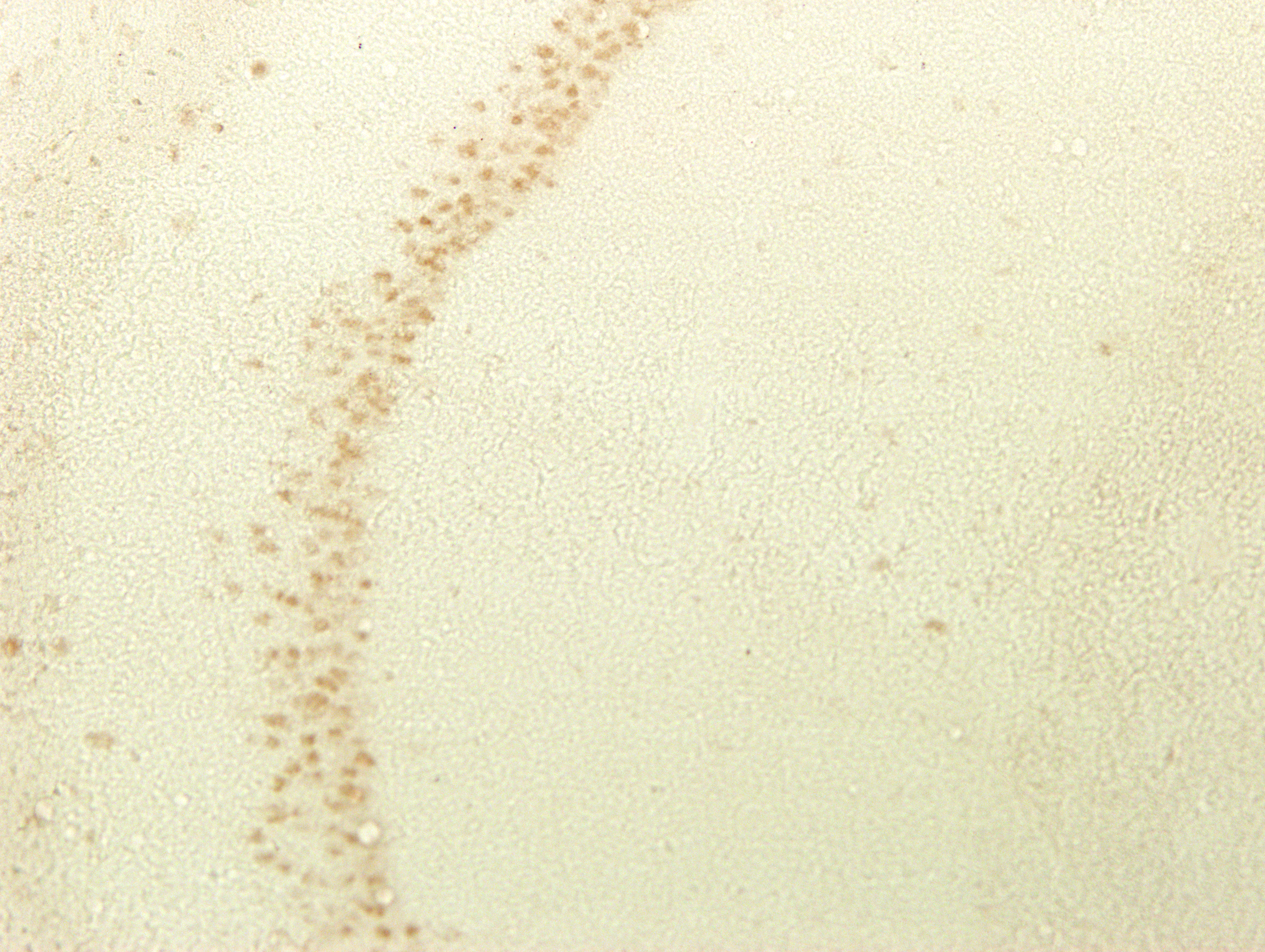

Supplement: Supplementary file 25 — Supplementary file25 (TIF 26795 KB) [file 43440_2022_430_MOESM25_ESM.tif]

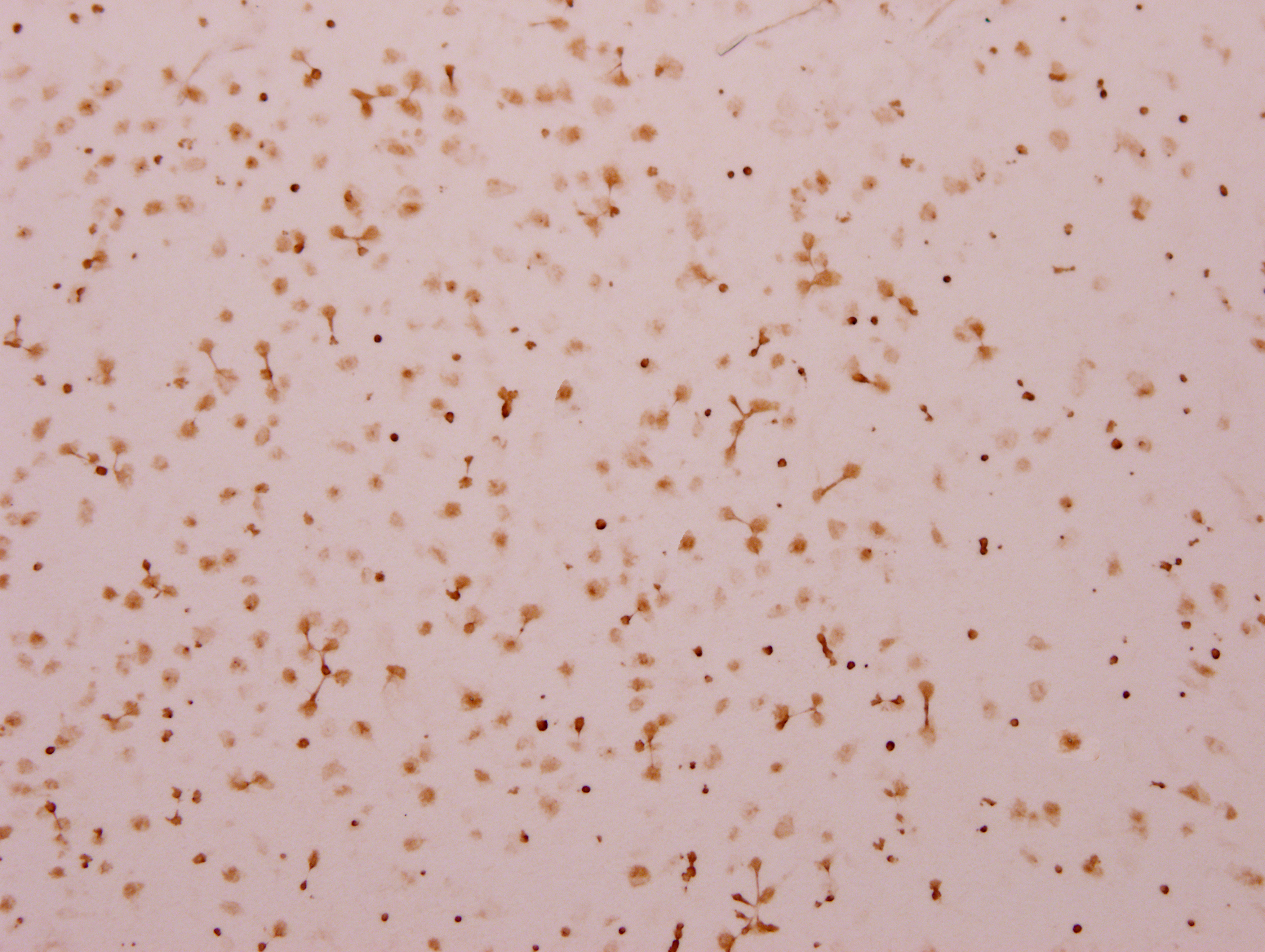

Supplement: Supplementary file 26 — Supplementary file26 (TIF 20646 KB) [file 43440_2022_430_MOESM26_ESM.tif]

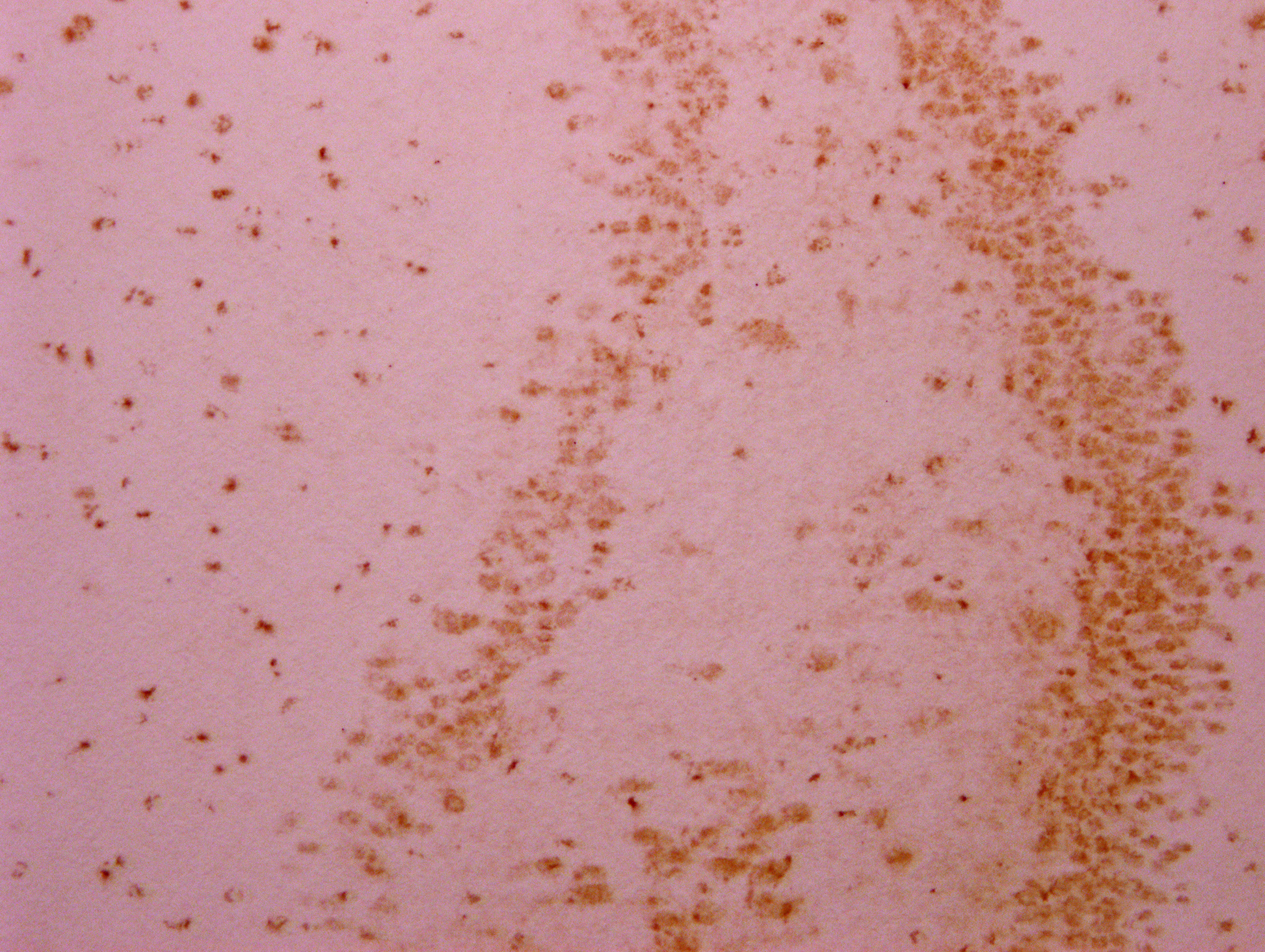

Supplement: Supplementary file 27 — Supplementary file27 (TIF 29526 KB) [file 43440_2022_430_MOESM27_ESM.tif]

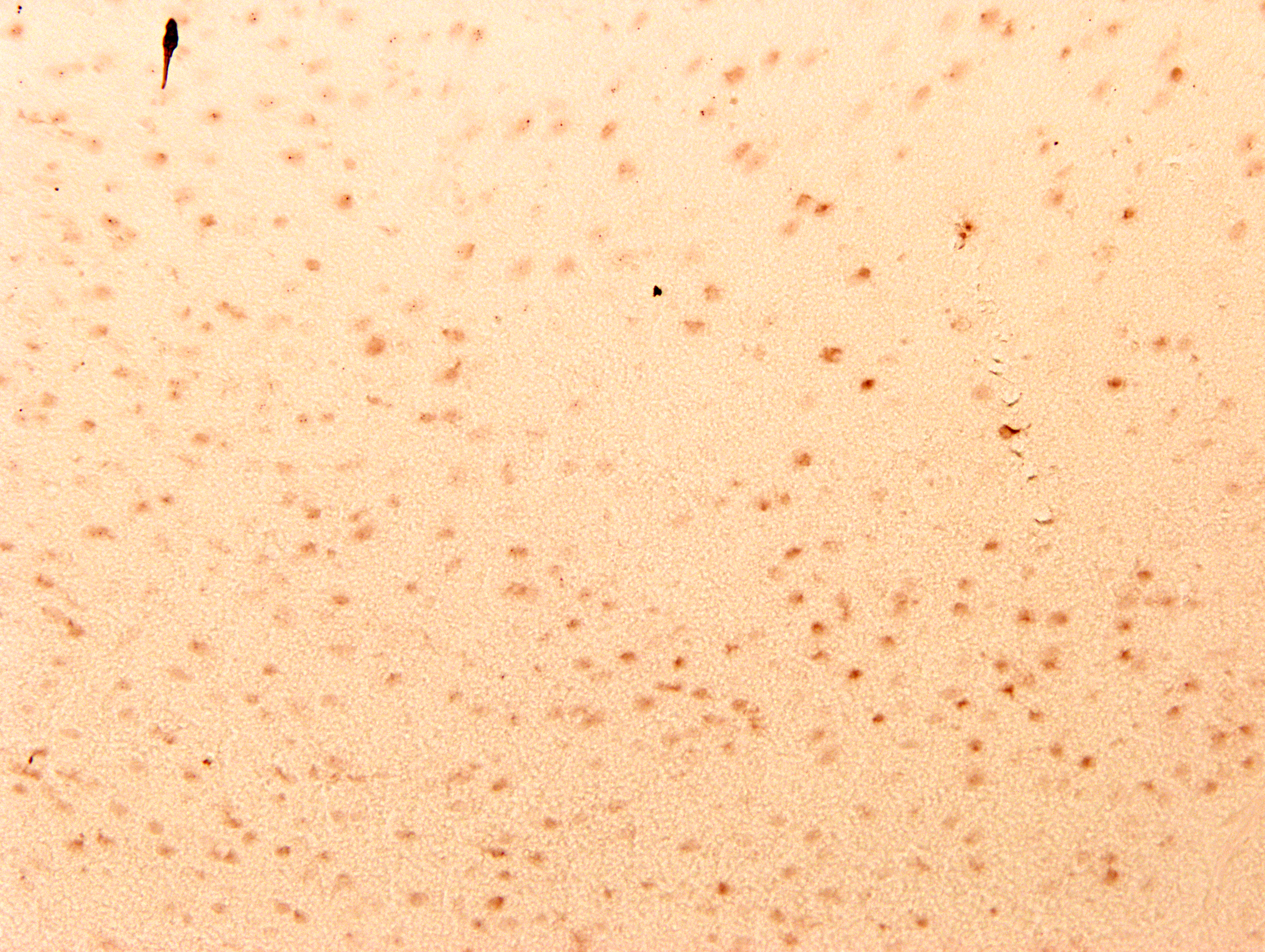

Supplement: Supplementary file 28 — Supplementary file28 (TIF 24610 KB) [file 43440_2022_430_MOESM28_ESM.tif]

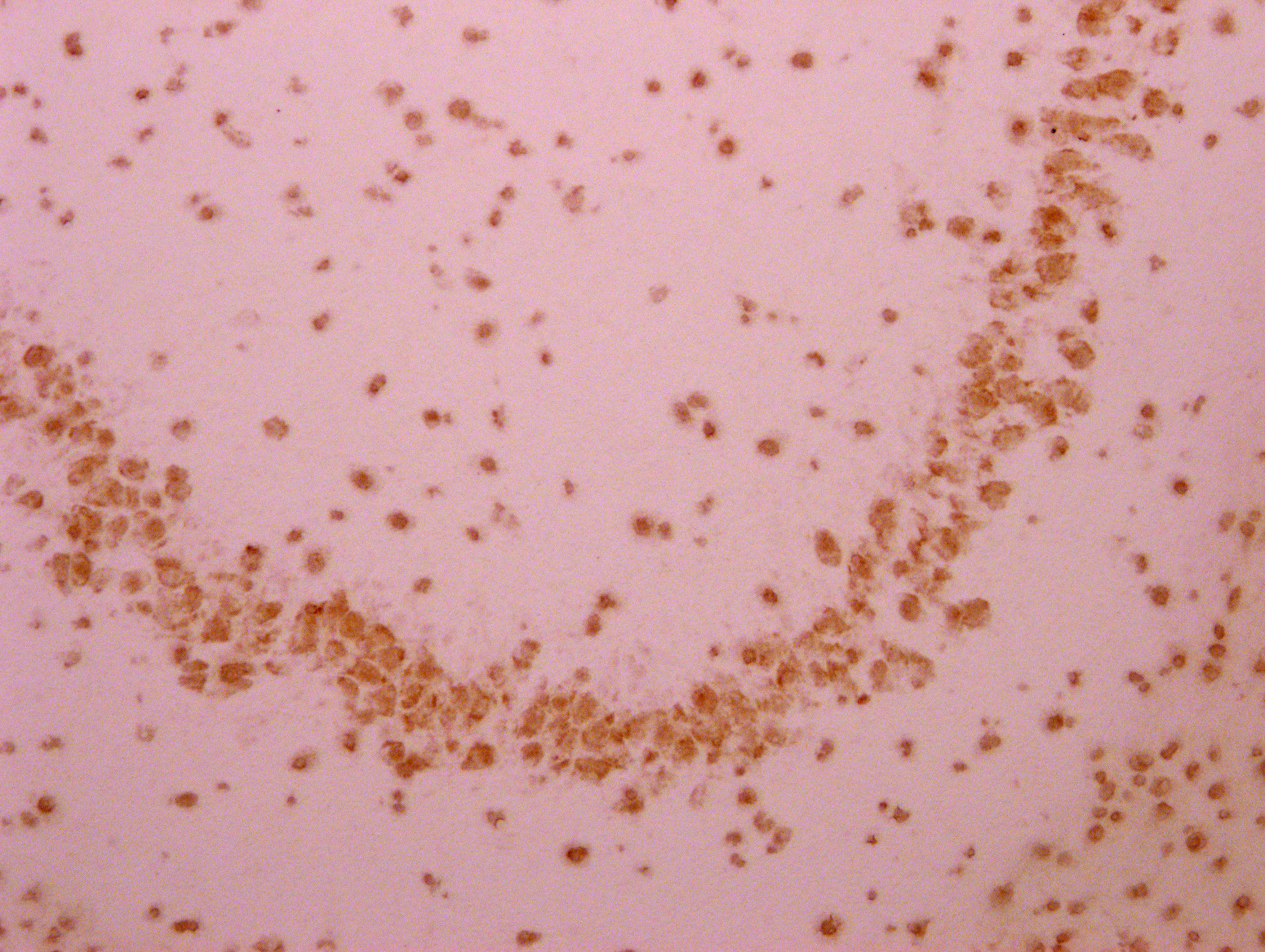

Supplement: Supplementary file 29 — Supplementary file29 (TIF 28392 KB) [file 43440_2022_430_MOESM29_ESM.tif]

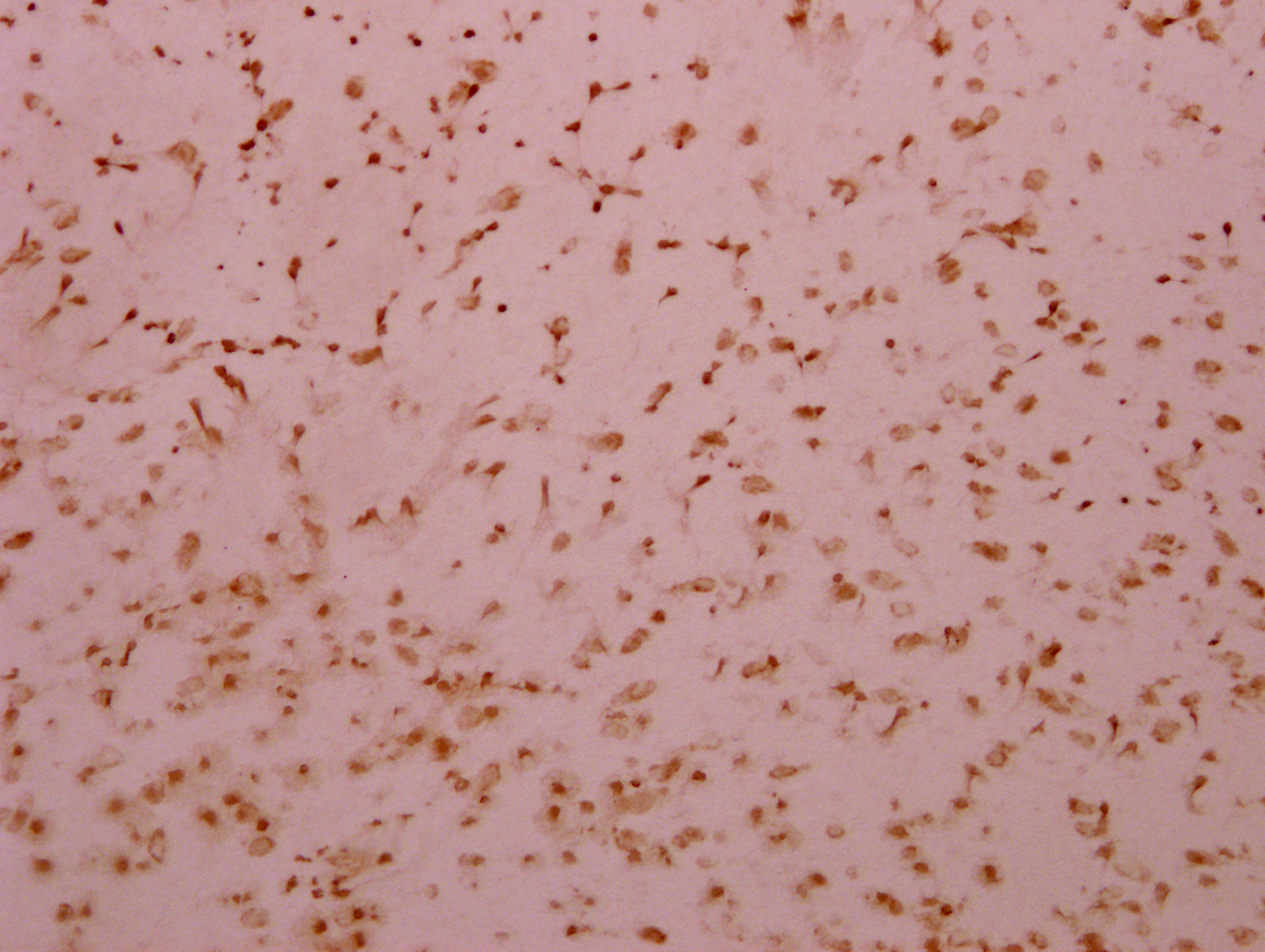

Supplement: Supplementary file 30 — Supplementary file30 (TIF 25155 KB) [file 43440_2022_430_MOESM30_ESM.tif]

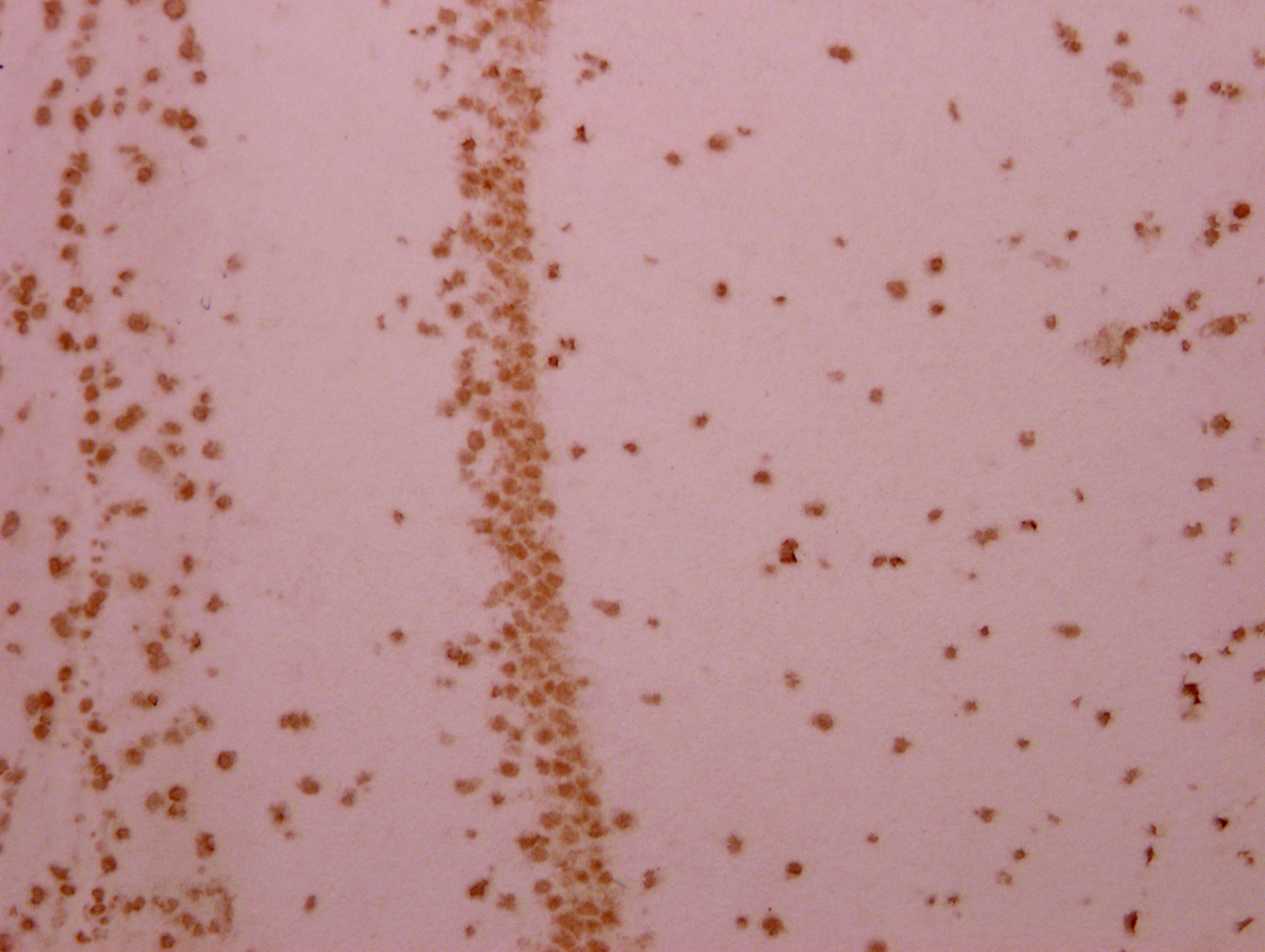

Supplement: Supplementary file 31 — Supplementary file31 (TIF 25582 KB) [file 43440_2022_430_MOESM31_ESM.tif]

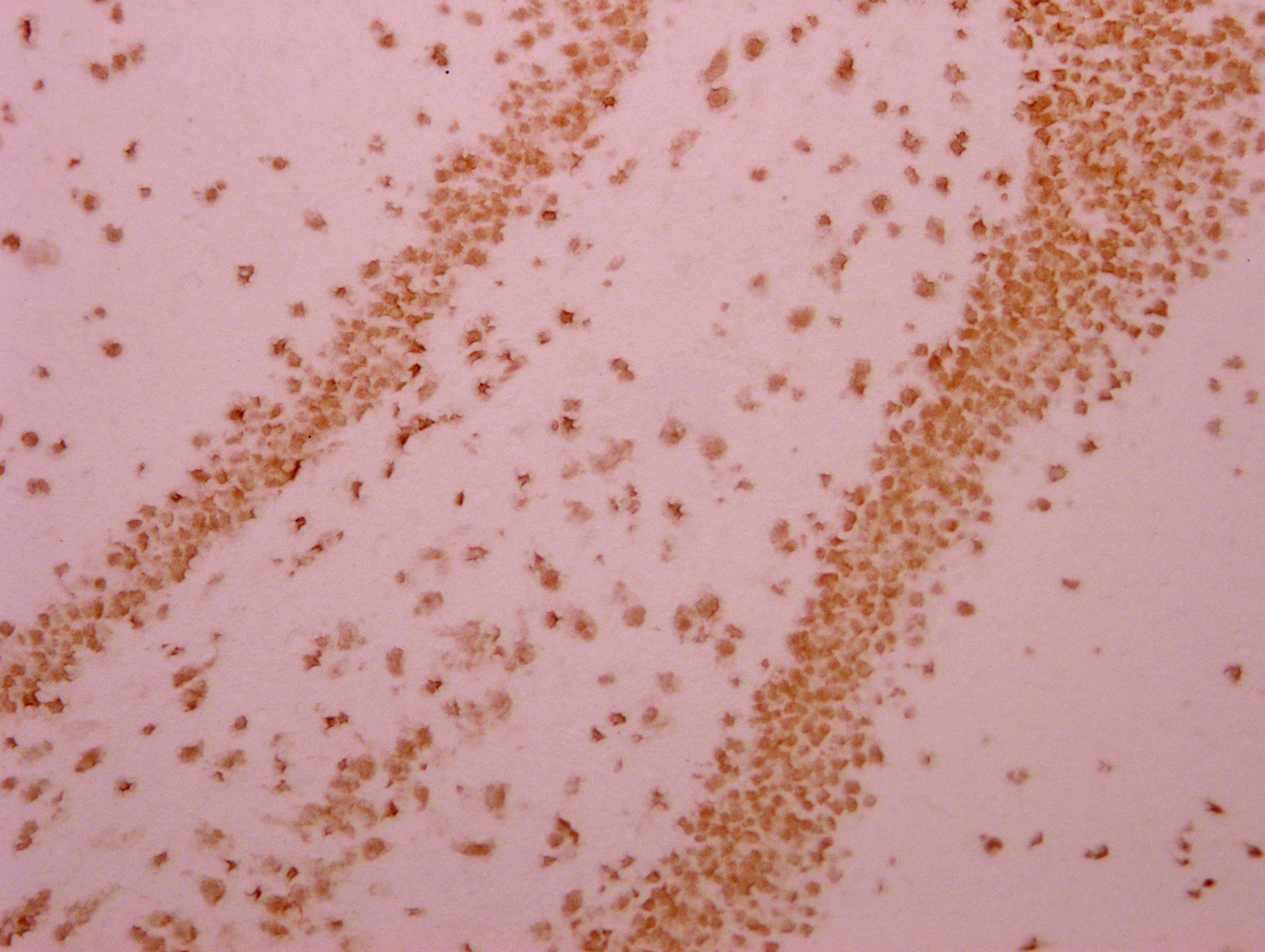

Supplement: Supplementary file 32 — Supplementary file32 (TIF 28313 KB) [file 43440_2022_430_MOESM32_ESM.tif]

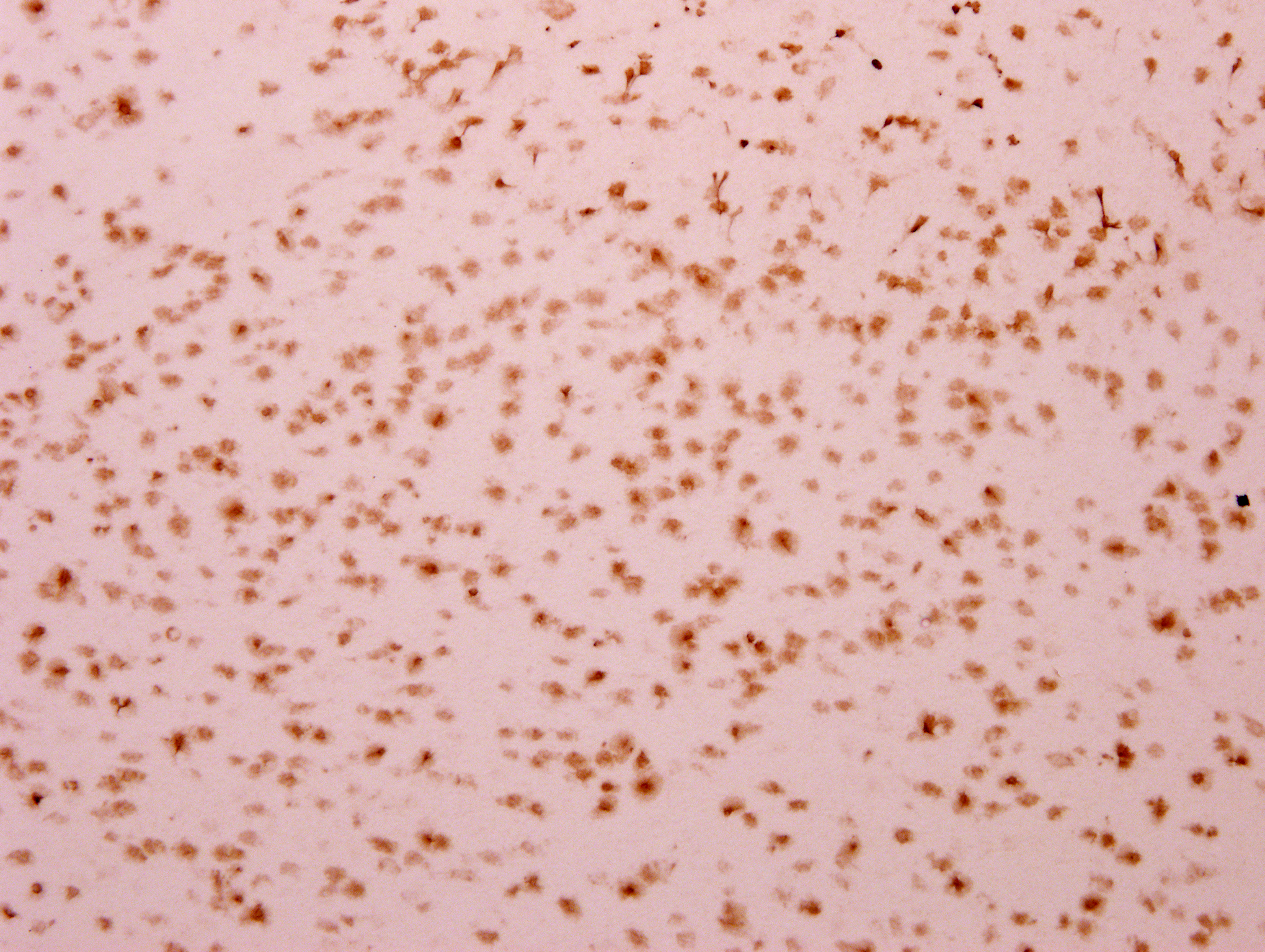

Supplement: Supplementary file 33 — Supplementary file33 (TIF 26329 KB) [file 43440_2022_430_MOESM33_ESM.tif]

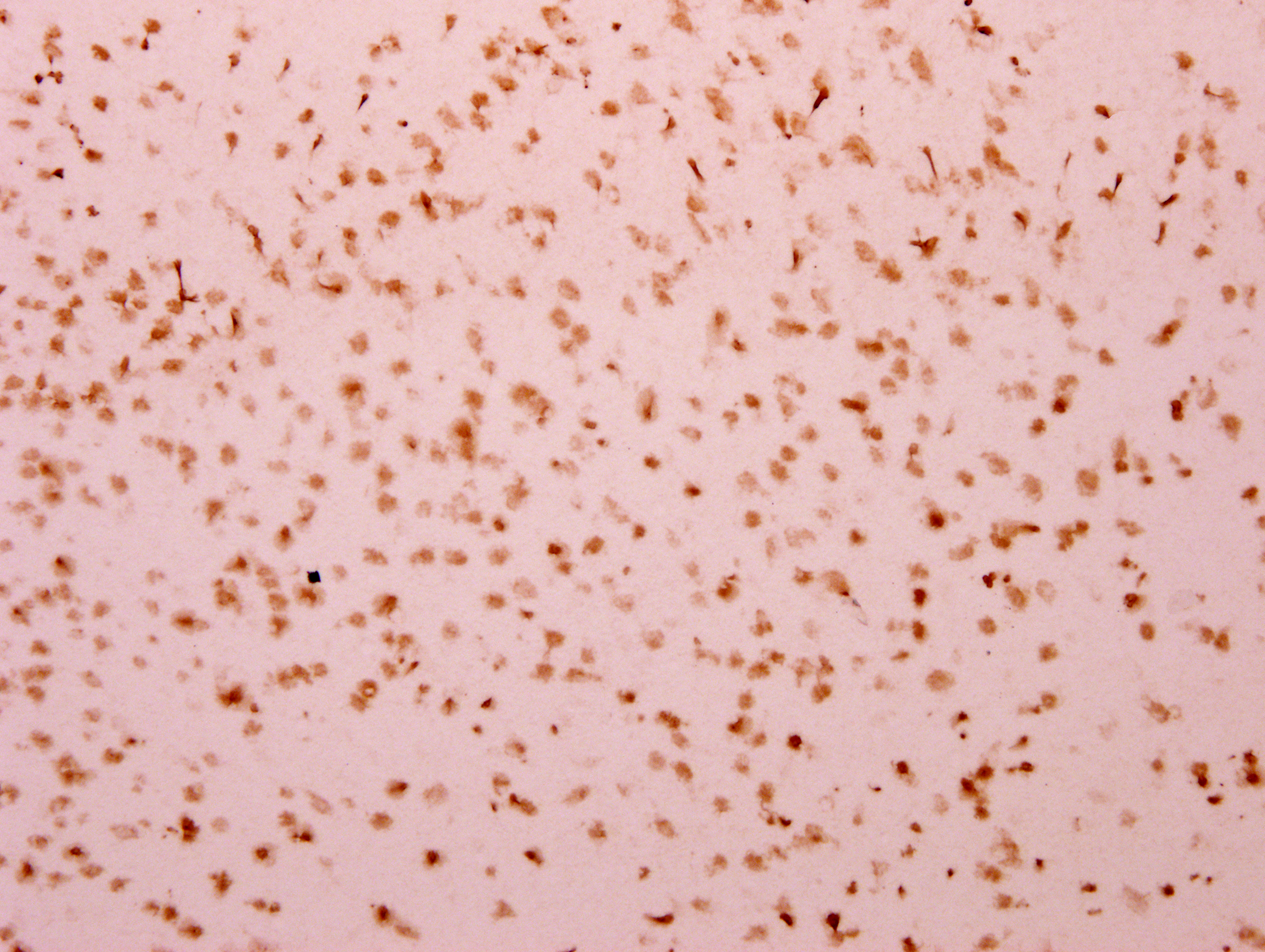

Supplement: Supplementary file 34 — Supplementary file34 (TIF 23250 KB) [file 43440_2022_430_MOESM34_ESM.tif]
